# Walking the Line: A Fibronectin Fiber-Guided Assay to Probe Early Steps of (Lymph)angiogenesis

**DOI:** 10.1371/journal.pone.0145210

**Published:** 2015-12-21

**Authors:** Maria Mitsi, Martin Michael Peter Schulz, Epameinondas Gousopoulos, Alexandra Michaela Ochsenbein, Michael Detmar, Viola Vogel

**Affiliations:** 1 Laboratory of Applied Mechanobiology, ETH Zurich, Zurich, Switzerland; 2 Institute of Pharmaceutical Sciences, ETH Zurich, Zurich, Switzerland; Ottawa Hospital Research Institute, CANADA

## Abstract

Angiogenesis and lymphangiogenesis are highly complex morphogenetic processes, central to many physiological and pathological conditions, including development, cancer metastasis, inflammation and wound healing. While it is described that extracellular matrix (ECM) fibers are involved in the spatiotemporal regulation of angiogenesis, current angiogenesis assays are not specifically designed to dissect and quantify the underlying molecular mechanisms of how the fibrillar nature of ECM regulates vessel sprouting. Even less is known about the role of the fibrillar ECM during the early stages of lymphangiogenesis. To address such questions, we introduced here an *in vitro* (lymph)angiogenesis assay, where we used microbeads coated with endothelial cells as simple sprouting sources and deposited them on single Fn fibers used as substrates to mimic fibrillar ECM. The fibers were deposited on a transparent substrate, suitable for live microscopic observation of the ensuing cell outgrowth events at the single cell level. Our proof-of-concept studies revealed that fibrillar Fn, compared to Fn-coated surfaces, provides far stronger sprouting and guidance cues to endothelial cells, independent of the tested mechanical strains of the Fn fibers. Additionally, we found that VEGF-A, but not VEGF-C, stimulates the collective outgrowth of lymphatic endothelial cells (LEC), while the collective outgrowth of blood vascular endothelial cells (HUVEC) was prominent even in the absence of these angiogenic factors. In addition to the findings presented here, the modularity of our assay allows for the use of different ECM or synthetic fibers as substrates, as well as of other cell types, thus expanding the range of applications in vascular biology and beyond.

## Introduction

The growth of new blood and lymphatic vessels from the pre-existing vasculature–angiogenesis and lymphangiogenesis respectively–serves essential functions in normal and pathological conditions, such as embryonic development, wound healing, cancer metastasis and inflammation [1–3]. Vascular Endothelial Growth Factors (VEGFs), acting via three tyrosine kinase receptors VEGFR1, VEGFR2 and VEGFR3, are the major regulators of (lymph)angiogenesis and VEGF signaling has been at the center of many therapeutic approaches targeting (lymph)angiogenesis[4,5]. While VEGF-A is considered to be the major angiogenic factor [6] and VEGF-C the lymphangiogenic one [7], there are studies showing VEGF-A inducing lymphangiogenesis [8–11] and conversely, VEGF-C promoting angiogenesis, [12–14], supporting a more complicated picture, with both growth factors involved in regulating different aspects of both angio- and lymphangiogenesis. However, how physical guidance cues, such as the presence of ECM fibers, affect the underlying regulatory signaling networks is largely unknown and thus leaves open the possibility that additional elements are involved in specifying the outcome of VEGF-A or -C stimulation. The major mechanism by which VEGF-stimulated (lymph)angiogenesis proceeds is via sprouting, a complex morphogenetic process initiated by tip cell selection and outgrowth [15]. The molecular mechanisms of sprouting have been and continue to be studied mainly in the context of angiogenesis. The current model holds that sprout growth is driven by a tip cell migrating along a VEGF-A gradient, and a body of stalk cells that proliferate to eventually form the lumen of the newly formed vessel [16]. A negative feedback loop between the Delta-like ligand 4 (Dll4)/Notch and VEGF-A/VEGFR2 signaling axes has been identified to underlie such cell behavior [17]. The mechanisms for sprouting of lymphatic vessels are less well understood, but there is some evidence that the same feedback loop between the VEGF-A/VEGFR2 and Dll4/Notch signaling pathways may be involved in lymphangiogenesis as well [18].

The classical view of a migrating tip cell and a body of stationary stalk cells that proliferate to form the lumen of the new vessel [15] has been challenged by recent findings [19,20], which show cell rearrangements during vessel sprouting, with cells in the stalk region migrating and often, overtaking the tip cell. The cell population within a sprout is highly heterogeneous and dynamic, with each cell having the potential to acquire any of a broad range of phenotypes between stalk and tip cell. This behavior correlates with differential adhesion between endothelial cells within the sprout but the causative factors that govern such individualistic response to VEGF stimulation remain unknown [20]. An interesting question is whether extracellular matrix (ECM) components may play a role in this process, especially if one considers the tight balance between cell-cell and cell-ECM adhesion that is required for such coordinated cell movement within a collective, as for example in epithelial intercalation and other instances of tissue morphogenesis [21,22]. Fibronectin (Fn) in particular, a major component of the basement membrane [23], plays a crucial role in vascular morphogenesis during development and tumor growth [24] and is closely linked with sprouting angiogenesis, as illustrated by the following examples. In retinal angiogenesis, the Fn matrix deposited by astrocytes guides the growth of the new vessels by providing a scaffold for integrin-mediated adhesion of the sprouting endothelial cells and by generating a matrix-bound VEGF gradient [25]. In another example, the sprouting of the interrenal vessel (IRV) from the dorsal aorta in zebrafish [26] requires the expression and deposition of Fn by the endothelial cells at the front of the sprouting vessel. The intersegmental vessels (ISV), a different set of vessels that sprout also from the dorsal aorta, grow along somite boundaries, which are enriched in Fn [27–29]. An interesting feature of ISV sprouting, which may be related to the nature of the substrate on which they grow, is that it proceeds via migration of a stream of endothelial cells connected to each other, in the absence of proliferation, and it results in the formation of a cord of cells, which becomes lumenized only at a later stage [29], in contrast to the classical migratory tip cell-proliferative stalk cells model [16]. A similar case of migration of streams of endothelial cells that form cord-like structures, initially lacking a lumen, has also been observed in the sprouting of the initial lymphatic cells from the cardinal vein during embryonic development, although the composition of the matrix on which they migrate has not yet been investigated [30]. In considering and interpreting a potential guiding role of Fn in those instances of endothelial cell streaming and sprouting, one should take into account the physical state of Fn within the ECM, which is fibrillar [31–34]. Indeed, angiogenesis in a 3D scaffold was shown to be driven by the *in situ* Fn fibrillogenesis carried out by endothelial cells, with the resulting network of Fn fibrils guiding the growth of the new vessels [35]. Similarly, Fn fibrillogenesis is upregulated during wound healing *in situ* by the newly formed vessels [36,37], thereby participating in the spatiotemporal coordination of the ongoing angiogenesis [38–41]. Highlighting the clinical importance of fibrillar ECM in sprouting, a recent study showed that although anti-angiogenic therapy through inhibition of VEGF signaling resulted in regression of tumor vessels, matrix-rich ‘sleeves’ were left behind and were readily repopulated by endothelial sprouts upon discontinuation of the therapy [42].

Such observations motivated us to develop a novel Fn fiber-guided (lymph)angiogenesis assay suitable to dissect the role of Fn, if presented in its fibrillar state, on the migration modes of endothelial cells during the early stages of (lymph)angiogenic sprouting. To achieve this goal, we combined a sprouting assay using beads coated with endothelial cells [43] with a stretch assay of fibrillar Fn [44]. The primary advantage of such an assay is the capability to address in a systematic and quantitative manner mechanistic questions pertaining to the regulatory role of Fn fibers in (lymph)angiogenesis at the single cell level. In a proof-of-concept study, we show here how early sprouting of lymphatic rather than blood vascular endothelial cells along Fn fibers is affected by VEGF-A, contributing novel knowledge to the field of sprouting lymphangiogenesis, which is not as extensively studied as angiogenesis. In the future, such knowledge may facilitate the design of more effective therapeutic approaches that can complement assays with high-throughput screening capability for test compounds.

## Materials and Methods

### LEC 2D and bead culture

Human dermal microvascular LEC from neonatal human foreskin were isolated as previously described [45] and were maintained in 2D culture on collagen type I (50 μg/ml, Advanced BioMatrix) coated plates in endothelial basal medium (Lonza) supplemented with 20% FBS (Gibco), 1% antibiotic/antimycotic solution (Gibco), 4 mM L-glutamine (Gibco), 25 μg/ml cAMP (Sigma) and 10 μg/ml hydrocortisone (Sigma) at 37°C complemented with 5% CO_2_. To generate LEC-coated beads, gelatin-covered cytodextran beads (Sigma) were used. The beads were hydrated, sterilized and incubated with LEC at a bead:cell ratio of 1:40. The suspension of beads and LEC was stirred for 2min every 30min for a total of 4h and incubated for another 48h, resulting in confluent bead coverage. A similar protocol was followed for the culture of human umbilical vein endothelial cells (HUVEC, purchased from Lonza and used until passage 8).

### Fabrication of the fiber-based (lymph)angiogenesis assay

Manually pulled Fn fibers were produced as previously described [44]. Briefly, Fn fibers were formed when a sharp tip was immersed and subsequently withdrawn from a droplet of a solution of 0.4 mg/ml Fn in PBS. The fibers were deposited onto transparent, elastic silicon sheets, which were either inserted into cell culture chambers, especially designed for microscopic observation, or were strained with a uniaxial stretching device and then secured in a clamping device, which allowed microscopic observation without strain release. When strain was applied, the fibers were deposited either parallel to the strain axis or in a grid. In the latter case, when the fibers parallel to the strain axis were stretched, the fibers vertical to the strain axis were relaxed. This design allows for having populations of fibers experiencing distinct strains in the same sample. The silicone sheets used as substrates for the fiber deposition were either passivated with 20 mg/ml BSA or coated with 20 μg/ml Fn prior to fiber deposition. If not otherwise specified, it will be assumed that the substrates were BSA passivated. For fluorescence microscopy, the fibers were pulled out of droplet of a Fn solution that contained 5% of Alexa633-succinimidyl ester labeled Fn.

### End point fluorescence confocal microscopy

Fully LEC covered beads were labeled with 2 μM cell tracker green (Invitrogen) or cell tracker red (Invitrogen) for 30min prior to the experiment, then added to the preparation of Fn fibers (Alexa633 labeled) (~ 1 bead/μl). The preparation was incubated for 24h at 37°C in imaging culture medium containing phenol red free endothelial basal medium (Lonza) supplemented with 20% FBS (Gibco), 1% antibiotic/antimycotic solution (Gibco), 4 mM L-glutamine (Gibco), 10 μg/ml hydrocortisone (Sigma) and 10 mM HEPES (Sigma) in the absence or presence of 50 ng/ml recombinant human VEGF-A_165_ or 200 ng/ml recombinant human VEGF-C (both growth factors from R&D Systems). Subsequently, the samples were imaged live at 37°C with a Leica SP5 laser scanning confocal microscope, with a 40x dry APO U-V-I (NA = 0.75) objective lens, using the Leica Application Suite Advanced Fluorescence 2.6.1.7314 software. To quantify the number of outgrowth events, the entire sample area containing fibers was defined by a rectangle and then covered with tiles in x and y direction, which were all imaged automatically. For all comparisons, only active beads were considered, i.e. beads that came in contact with at least one fiber and gave rise to at least one fiber outgrowth event. To compare fiber outgrowth under different experimental conditions (in the presence or absence of VEGF-A / VEGF-C, or on fibers experiencing different strain values), the number of outgrowth events per fiber was counted for all active beads in each sample. For example, for a bead that came in contact with one fiber and gave rise to two outgrowth events (one in each direction) a value of 2/1 = 2 outgrowth events per fiber was calculated. In a different type of analysis, employed to compare outgrowth to the fibers versus the underlying 2D substrate in the same experimental setup, we calculated the percentage of active beads which gave rise to 2D outgrowth in addition to the fiber outgrowth (beads that came in contact with a fiber were never observed to give rise to a 2D outgrowth event but not fiber outgrowth). To confirm the capacity of the underlying 2D substrate to sustain outgrowth, the percentage of beads that did not come in contact with a fiber but gave rise to a 2D outgrowth event was also calculated.

### Time-lapse video microscopy

Samples were prepared as described for fluorescence microscopy, using unlabeled LEC and Fn fibers. Following a 6h incubation time at 37°C, the samples were transferred into the incubation chamber of an Olympus CellR IX81 epi-fluorescence microscope (37°C, 5% CO_2_, humidified) with a 10x 0.3NA Ph1 UPLANFL objective lens with the CellR 3.0 software (Olympus) and were imaged under phase contrast every 10min for 48h to 72h. For every time point, four z-planes were acquired, and the best focused z-plane was selected for post-acquisition image analysis. Outgrowth events were classified into two categories: single and collective cell migration. A single event starts from the moment a single cell emerges from the bead. After the cell body has fully emerged, the cell severs all connections with the bead and migrates along the Fn fiber as a single cell, until it leaves the field of view, becomes incorporated into another bead or returns to the bead of origin, undergoes division or apoptosis, or joins an adjacent cell cluster. The fate of the cell after it completely leaves the bead of origin does not affect its classification as a single migrator. In contrast, collective cell migration begins with a single cell emerging from the bead followed by trailing cells that together move along the fiber as a unit without disrupting cell-cell connections. The cells comprising the collective event can be arranged in a linear fashion or form a cluster around the fiber. To quantitatively analyze the migration patterns of the outgrowing cells, the paths of single cells or leading cells of collective outgrowth events were recorded by manually tracking in the image sequences the cell centers using the Manual Tracking plugin from ImageJ (NIH).

### Whole mount immunofluorescence staining

Neonatal mice (P6) were killed by decapitation and their diaphragm was collected and fixed in 4% formaldehyde. The tissue was blocked and permeabilized overnight in PBS containing 5% donkey serum and 0.1% Triton-X (Sigma). Antibodies used were rabbit anti-LYVE-1 (11–034, AngioBio), goat anti-Fn (N-20, Santa Cruz Biotechnology inc.) and AlexaFluor 488 nm and 594 nm-conjugated secondary antibodies (Invitrogen). Tissues were mounted in Vectashield (Vector) for confocal imaging. Whole-mount z-stack images were acquired with a LSM 710 FCS confocal microscope with a 10x 0.3NA EC Plan-Neofluar objective lense with the ZEN 2010 software (all Zeiss) and processed with Photoshop CS5 (Adobe) and ImageJ software (NIH). Mice used in this study were bred and housed in the animal facility of ETH Zurich. Experiments were performed in accordance with the animal protocol 131/2014 approved by the local veterinary authorities (Kantonales Veterinäramt Zürich).

### Statistical analysis of outgrowth events and migration parameters

When we analyzed beads that gave rise to multiple outgrowth events during the experiment, we observed that events originating from the same bead belonged to the same outgrowth type (single or collective) more often than predicted by a fully random outgrowth model (S1 Fig). This suggests that the factors determining the mode of outgrowth (single or collective) act at the level of the bead and the behavior of cells originating from the same bead is to some extent correlated. To account for such correlations, we counted the number of beads giving rise to single or collective outgrowth, rather than the number of individual events. Similarly, the migration parameters of the outgrowing cells (displacement and cumulative distance values) were averaged at the level of beads: values for outgrowth coming from the same bead and belonging to the same type–single or collective–were averaged and only this average was considered for the analysis. Evidence for such correlations came from the fact that the range of values for outgrowth events originating from the same bead and belonging to the same type (single or collective) was significantly smaller compared to the range of values across all beads (S2 Fig). For the statistical analysis of displacement and cumulative distance values, the normality of the data was tested by QQ plots (plotting the quantiles of the data population against the quantiles of the normal distribution) with the expectation that data from a population following a normal distribution should fall into a line. The data from all four populations tested in this study fit reasonably well to the normal distribution, supporting the usage of statistical tests that assume normality (S3 Fig). Accordingly, we used robust linear regression models to asses statistical significance of differences between displacement and cumulative distance values, as described in detail in S4–S8 Figs). Furtermore, such models tolerate well small deviations from normality.

### Sample embedding in collagen gels for immunofluorescence microscopy

To preserve the relative position of beads on the Fn fibers together with the outgrown cells at the end of the assay, the samples were embedded in collagen gels prior to immunostaining. Briefly, samples were prepared as described for fluorescence microscopy, using unlabeled LEC or HUVEC and Fn fibers. Following a 6h incubation time at 37°C, the samples were fixed with 4% formaldehyde and subsequently covered with 4 mg/ml collagen. The collagen was left to polymerize at 37°C for 20 min and subsequently was washed for 2h with PBS. The samples were additionally fixed for 15min with 4% formaldehyde, washed with 0.1M glycine for 15min and blocked with 10% donkey serum in PBS for 1h. Antibodies used were anti VE-cadherin (R&D Systems) and Alexa Fluor 488 conjugated secondary antibody (Invitrogen). Cell nuclei were counterstained with Hoechst bisbenzimide (Sigma-Aldrich) and the actin cytoskeleton with Alexa Flour 549 conjugated phalloidin (Invitrogen). Stained specimens were examined with a Leica SP5 laser scanning confocal microscope, with a 40x dry APO U-V-I (NA = 0.75) objective lens, using the Leica Application Suite Advanced Fluorescence 2.6.1.7314 software and the images were processed by ImageJ (NIH).

### Electron microscopy

Samples were prepared as described for fluorescence microscopy, using unlabeled HUVEC and Fn fibers. Following a 6h incubation time at 37°C, the samples were washed once with PBS, high pressure frozen, freeze substituted in acetone and embedded in Epon. Ultrathin sections were observed with transmission electron microscopy (TEM), using a FEI Morgagni 268 TEM (100 kV).

## Results

### Fn fibers colocalize with developing, LYVE-1 positive lymphatic vessels in the muscular region of neonatal mouse diaphragms

**Fig 1:**
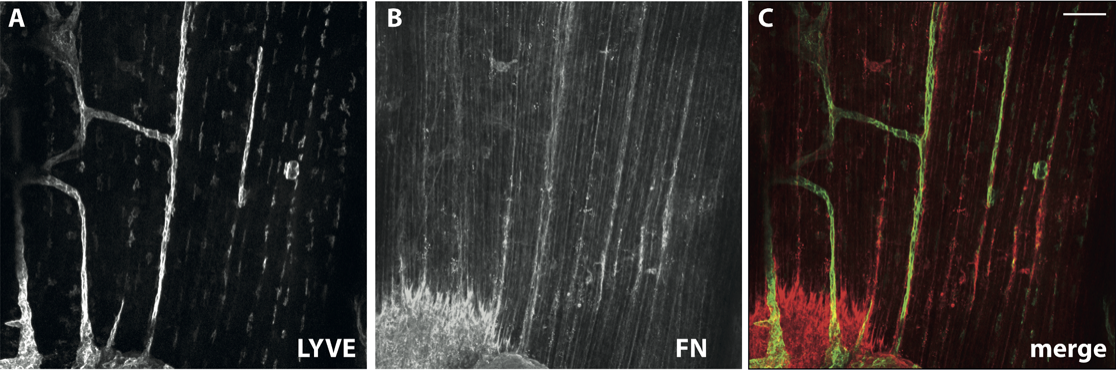
Lymphatic vessels colocalize with Fn fibers *in vivo*. Whole mount staining of neonatal mouse diaphragm demonstrated colocalization of LYVE-1 positive lymphatic vessels (A) with a distinct fibrillar arrangement of Fn (B). The merged image of LYVE-1 (green) with Fn (red) staining is shown in (C). The images in (A) and (B) are shown in gray scale for more clarity. Maximum intensity projections of confocal microscopy images are shown. Scale bar represents 100 μm.

Since most previous studies have focused on the role of Fn in angiogenesis [24–29] but not lymphangiogenesis, we immunostained lymphatic vessels in the muscular region of neonatal mouse diaphragms and found that Fn fibers, arranged in a parallel manner, do indeed colocalize with developing, LYVE-1 positive lymphatic vessels (Fig 1). This observation supports the hypothesis that Fn fibers within the ECM could act as tracks to guide the sprouting of new lymphatic vessels as well.

### Capturing the initial steps of sprouting (lymph)angiogenesis at the single cell level with a novel Fn fiber-based assay

**Fig 2:**
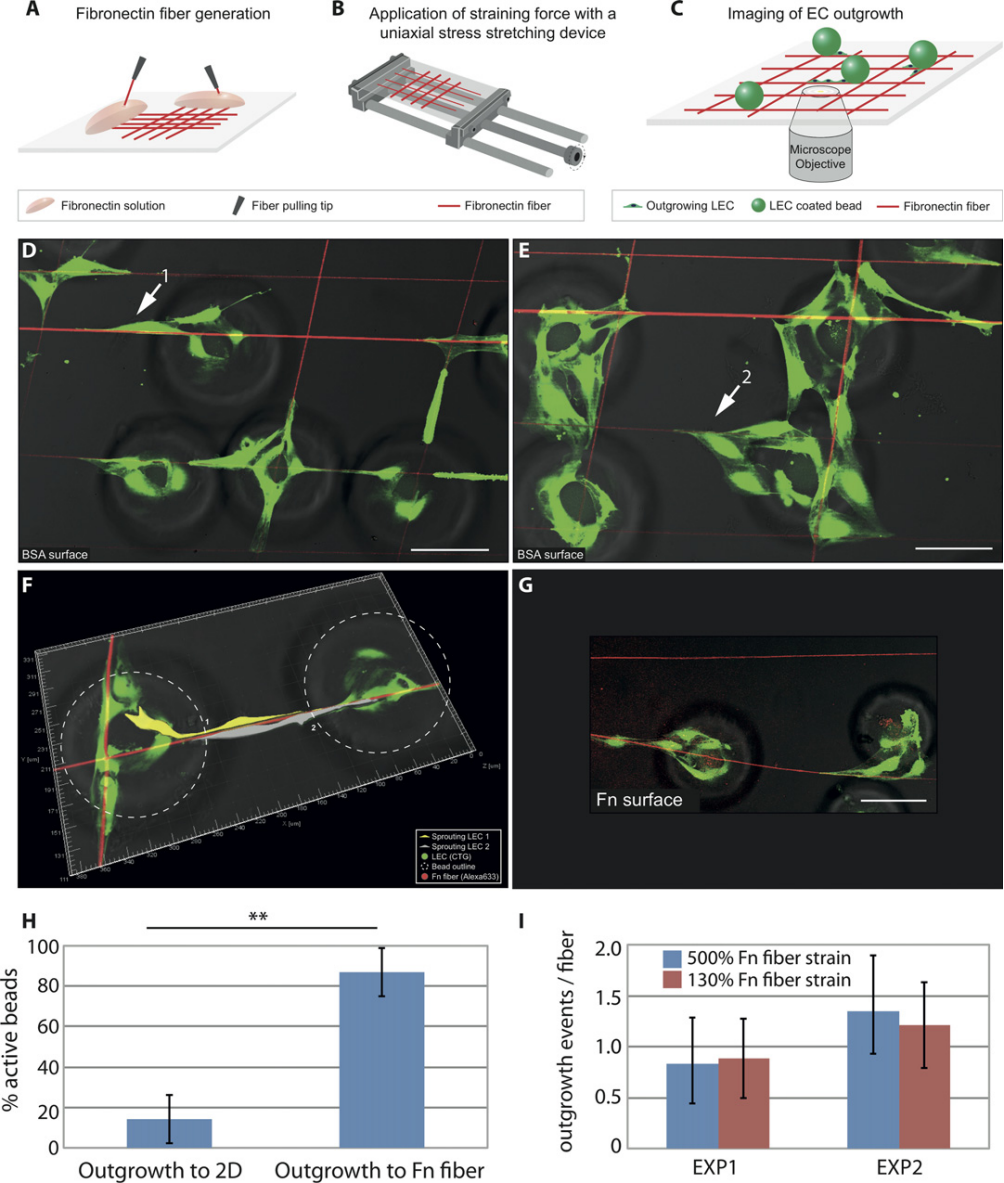
LEC outgrowth occurs preferentially along Fn fibers. (A-C) Schematic representation of the Fn fiber-guided (lymph)angiogenesis assay mimicking the *in vivo* situation, where the experimental setup allows for directional cell outgrowth. Fn fibers are produced by manually pulling them from a droplet of a Fn solution and are deposited on chemically inert and transparent silicone sheets (A), which allow stretching them to defined mechanical strains (B). The fibers are incubated with LEC coated beads and outgrowth events are monitored microscopically (C). (D-I) Beads cultured with cell tracker green (CTG) labeled LEC (green) were allowed to interact with Alexa-633 labeled Fn fibers (red) and the samples were imaged after 24h with a fluorescence confocal microscope. (D, E) The underlying 2D substrate was passivated with 20 mg/ml BSA, and the samples contained Fn fibers experiencing two distinct strain values: 500% for the horizontal and 130% for the vertical Fn fibers. Two classes of outgrowth events are marked by arrows: single cell outgrowth (1) and collective outgrowth (2). (F) 3D reconstruction of two interacting outgrowing LEC from adjacent beads onto the fiber between them (pseudo-colored in gray and yellow). (G) The Fn fibers were deposited on substrates that were pre-coated with 20 μg/ml Fn experiencing preferential outgrowth to the Fn fiber. (H) Samples were prepared as in (G). The entire sample area was imaged and LEC outgrowth events were analyzed, demonstrating that for the majority of beads LEC preferred outgrowth to Fn fibers over the Fn coated 2D substrate, if the beads were in contact with a Fn fiber. To calculate the depicted mean ± SD values, 17 to 40 beads per condition were analyzed. Chi-square p-value < 0.0001. (I) LEC outgrowth events onto Fn fibers of stretched (500%) vs. relaxed (130%) states were counted demonstrating no statistically significant difference in two independent experiments (p-values from ttest: 0.8 for EXP1 and 0.2 for EXP2). The entire area was imaged and to calculate the depicted mean ± SD values, 11 to 47 beads per condition were analyzed. Scale bar represents 100 μm.

To explore how interactions with Fn fibers impact the migration patterns of lymphatic endothelial cells during the initial phases of sprouting, we set up an assay whereby oriented Fn fibers could serve as substrate to support outgrowth of lymphatic endothelial cells as follows: Fn fibers were pulled from concentrated droplets of a Fn solution and deposited on silicone substrates in a parallel fashion [44,46,47]; subsequently, LEC cultured to confluence on gelatin-coated microcarrier beads were brought in contact with the pulled Fn fibers, providing a quiescent cell layer that can act as a source of sprouting cells. Since the silicone substrates used to deposit the Fn fibers are transparent and compatible with microscopy, the LEC-Fn interactions can be monitored over time at the single-cell level (Fig 2A–2C).

Following a 24h incubation time of the LEC-coated beads with the Fn fibers, several outgrowth events from the beads to the fibers were observed (Fig 2D and 2E; LEC labeled in green and Fn in red). Examples of such events include single cell protrusions along a fiber (Fig 2D arrow 1) and multicellular clusters on and around a fiber (Fig 2E arrow 2). A 3D reconstruction of two LEC outgrowth events from two adjacent beads is shown in Fig 2F, documenting the outgrowth and migration of LEC along the Fn fiber.

These results show that LEC at the base of beads that came in contact with a fiber are given guidance cues to initiate outgrowth. To test whether such guidance cues are unique for fibrillar Fn, we coated the substrate surface with Fn prior to fiber deposition and compared the competition of LEC outgrowth onto the flat Fn-coated surface *versus* along the Fn fiber (Fig 2G). When considering all the beads that came in contact with a fiber and led to LEC outgrowth (active beads) a strong preference for fiber outgrowth was observed (Fig 2H): 87% of the active beads showed outgrowth exclusively to the Fn fibers, whereas only 14% of active beads showed outgrowth to the surface-adsorbed Fn (2D outgrowth) in addition to fiber outgrowth. As an internal control, the percentage of the beads that did not contact a fiber but resulted in at least one 2D outgrowth event was 56% (data not shown), ensuring that the preference for fiber outgrowth is not a result of the inability of the Fn-coated 2D substrate to sustain cell outgrowth. These results clearly demonstrate the importance of the fibrillar organization of Fn to guide LEC outgrowth from a confluent cell layer (in the case of our experimental setup this layer is provided by an LEC-coated bead, but in a more physiologically relevant setting it could be the quiescent endothelial layer of a vessel prior to sprouting). However, it is not clear from these data, whether it is the conformation of individual Fn molecules within the fiber that is responsible for the strong outgrowth cues or if additional features contribute, such as the density of Fn molecules on the fibers and/or the fibrillar geometry.

### Fn fibers stretched to different strains can all support LEC outgrowth

Fn conformation plays a pivotal role steering the biological function of the protein by regulating the exposure of various binding sites and one of the mechanisms that cells employ to regulate Fn conformation is by exerting mechanical forces on the Fn fibers within the ECM [48–50]. Since the Fn fibers can either be deposited on glass or on stretchable substrates, our assay provides the capability to mechanically stretch the Fn fibers prior to bead seeding (Fig 2B). In this way, we can externally adjust the fibers to a broad range of mechanical strains and corresponding molecular conformations, similar to those found in native ECM [48] and test their respective ability for LEC outgrowth. Our data show that Fn fibers stretched to different strains (500% for horizontal and 130% for vertical fibers shown in Fig 2D and 2E) can support LEC outgrowth independent of their mechanical strains (Fig 2I). It appears then, that at least mechanically induced alterations of Fn conformation, which could destroy binding sites on the surface of the fibers [51,52] do not affect the capacity of the fibers to stimulate LEC outgrowth, suggesting that Fn fibers do not lose their capacity to act as guiding tracks for newly sprouting vessels in different physiological settings, even in situations where the ECM is being stretched to various degrees (for example, contracting wounds [53,54] and inflamed lymphatic nodes [55]).

### VEGF-A, but not VEGF-C stimulates the collective outgrowth of LEC, but not of HUVEC

**Fig 3:**
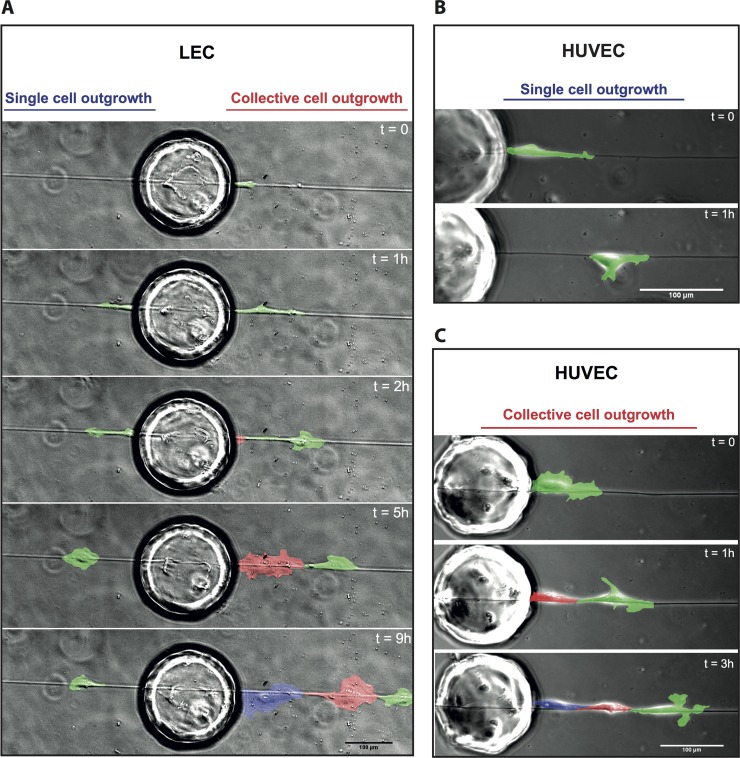
Outgrowth of LEC and HUVEC on Fn fibers is either single or collective. LEC (A) or HUVEC (B,C) coated beads incubated with Fn fibers for 6h and then observed by phase contrast video-lapse microscopy. t = 0 denotes the beginning of the first outgrowth event. (A) Time series showing two LEC events initiated from one bead (each cell pseudo-colored with a different color for clarity). Images temporally depict a single outgrowth event to the left of the bead and a collective event initiated by a leading cell to the right (also refer to S1 Video). (B) Time series showing one HUVEC single outgrowth event (pseudo-colored in green) from one bead (also refer to S2 Video). (C) Time series showing one HUVEC collective outgrowth event (each cell pseudo-colored with a different color for clarity) from another bead (also refer to S3 Video). Scale bar represents 100 μm.

In a first proof-of-concept study, we exploited the capability of our assay to observe the dynamics of cell outgrowth to Fn fibers in real time and at the single cell level. Cell outgrowth dynamics were monitored over 48h, every 10min, by phase contrast microscopy, as the LEC sensitivity prevented continuous fluorescence illumination. Cell migration observed in all the time-lapse videos taken can be categorized into two types of cell movement: single and collective outgrowth events. Representative examples of these types of cell movements for LEC are captured in Fig 3A and S1 Video, where a time sequence of two outgrowth events from one bead to a Fn fiber is shown. To the left, one single cell migrates away from the bead along the fiber. To the right of the bead, and at about the same time, an instance of collective cell motion is initiated, whereby several cells (each depicted by a different color for clarity), guided by a leading cell, migrate away from the bead in an aligned and connected manner.

To investigate whether such cell behavior is specific for LEC, we repeated the experiments using HUVEC and we observed similar types of outgrowth. Examples of single and collective cell outgrowth for HUVEC are shown in Fig 3B and Fig 3C and in S2 and S3 Videos respectively.

**Fig 4:**
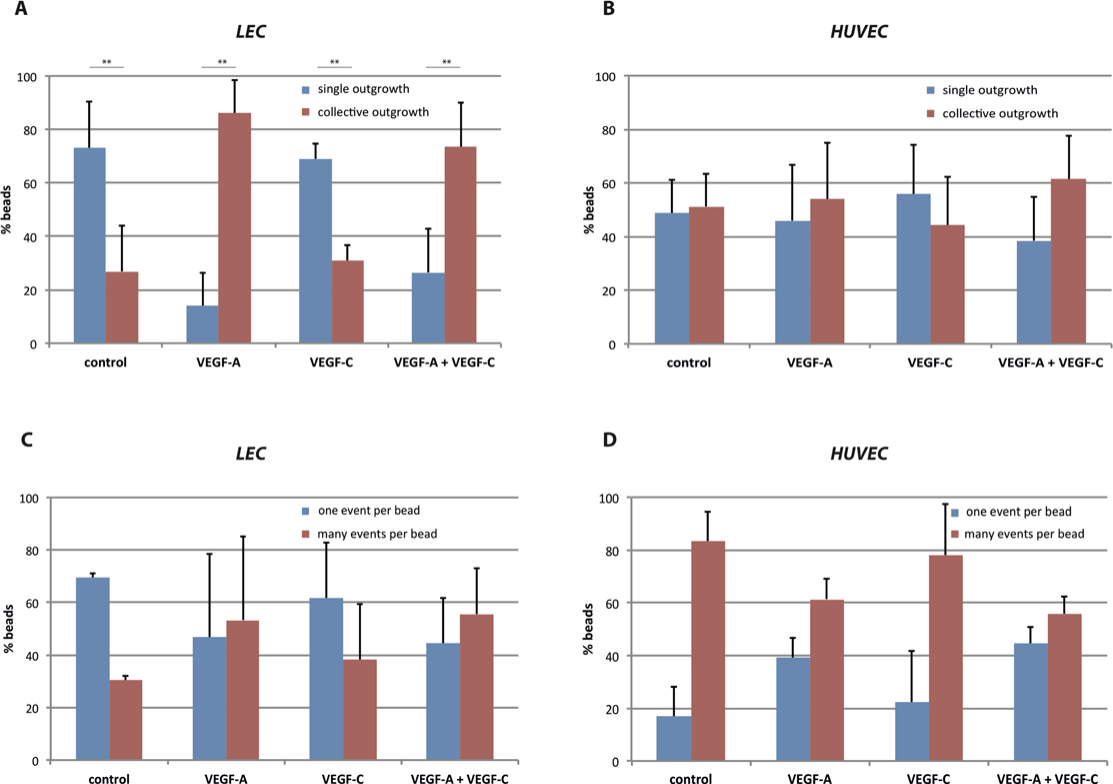
The presence of VEGF-A favors collective LEC outgrowth but has no effect on the mode of HUVEC outgrowth. (A,B) Bar graphs quantifying for LEC (A) and HUVEC (B) the % number of beads with single cell outgrowth (blue) and collective cell outgrowth events (red), counted in the absence or presence of 50 ng/ml VEGF-A, 200 ng/ml VEGF-C, or 50 ng/ml VEGF-A plus 200 ng/ml VEGF-C in the medium. (C,D) Bar graphs quantifying for LEC (C) and HUVEC (D) the % number of beads that gave rise to only one outgrowth event (blue) or multiple events (red) during the experiment, counted in the absence or presence of 50 ng/ml VEGF-A, 200 ng/ml VEGF-C, or 50 ng/ml VEGF-A plus 200 ng/ml VEGF-C in the medium. Statistical significance was tested with the chi-square test (S1 Table). p-values: LEC single vs. collective <0.0001, LEC one vs. multiple events per bead = 0.0454, HUVEC single vs. collective = 0.502, HUVEC one vs. multiple events per bead = 0.0356. ** p-value < 0.0001.

Since VEGF-A and VEGF-C are major drivers of both angiogenesis and lymphangiogenesis [56], we next quantified their effect on the outgrowth activity of LEC and HUVEC as described in detail in the Methods section and S1 Fig For LEC (Fig 4A), in the absence of any growth factors, the majority of outgrowth events were single (73% single, 27% collective), whereas addition of 50 ng/ml VEGF-A increased significantly the percentage of beads giving rise to collective outgrowth (14% single, 86% collective). Interestingly, addition of 200 ng/ml VEGF-C, an amount sufficient to induce LEC outgrowth in a conventional sprouting assay [18], had no effect on the mode of LEC outgrowth to the Fn fibers (69% single, 31% collective), and addition of both growth factors showed a pattern similar to that of VEGF-A alone (26% single, 74% collective). In contrast to LEC, addition of the same amount of VEGF-A and/or VEGF-C did not have a statistically significant effect on the outgrowth mode of HUVEC (Fig 4B).

In addition to the mode of outgrowth (single or collective), we also monitored the percentage of beads that gave rise to multiple events during the experiment (Fig 4C and 4D). Addition of VEGF-A and/or VEGF-C had no statistically significant effect of the number of events per bead, at least at the 0.01 level, for neither LEC (Fig 4C) nor HUVEC (Fig 4D). It is interesting to notice that for HUVEC, one collective outgrowth event often lasted uninterrupted through the entire time-lapse video (S4 Video), whereas several single outgrowth events could occur during the same period of time (S5 Video). In addition to the percentages shown in Fig 4, the actual number of beads, along with the statistical analysis, is shown in S1 Table.

In summary, we observed that VEGF-A stimulated the collective outgrowth of LEC onto Fn fibers, while this was not observed with VEGF-C alone, or with HUVEC.

### Collective outgrowth exhibits higher persistence of motion directionality compared to single cell outgrowth

**Fig 5:**
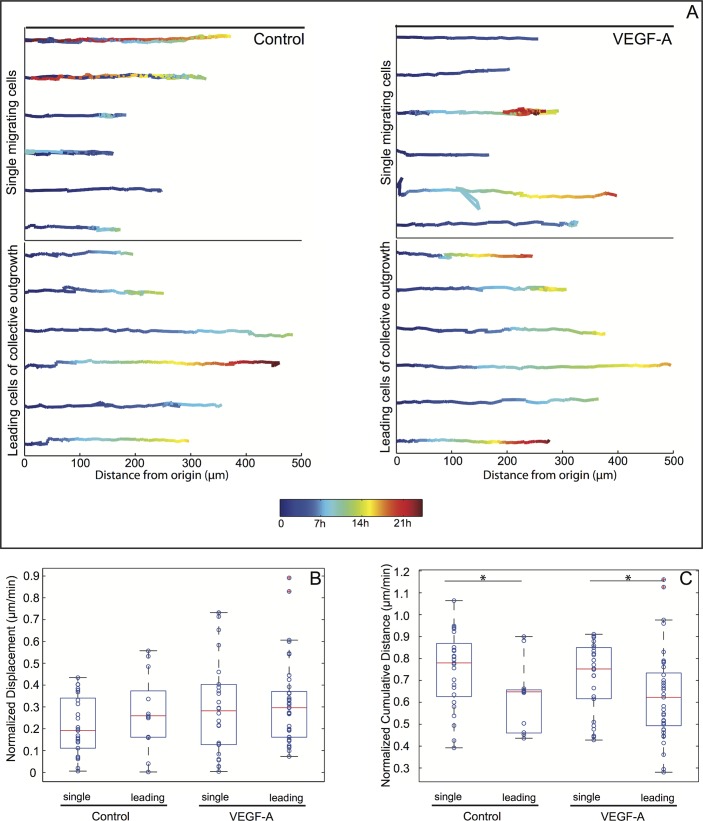
Leading cells of collective LEC outgrowth events exhibit higher motion persistence. LEC coated beads were incubated with Fn fibers for 6h and then observed with phase contrast video-lapse microscopy. (A) Representative trajectories of cells migrating along Fn fibers, either as single cells (upper panel) or as leading cells of collective outgrowth events (lower panel), in the absence (left) or presence (right) of 50 ng/ml VEGF-A. The cell tracks shown have been normalized with respect to their origin, as well as the fiber angle. The color denotes time, from the beginning of the track (blue) until 24h (red). From all the cell trajectories analyzed, normalized displacements (B) and cumulative distances (C) travelled by the cells are plotted as box plots, where the central mark is the median (16 to 50 beads per conditions were analyzed), the edges of the box are the 25^th^ and 75^th^ percentiles, the whiskers extend to the most extreme data points not considering outliers (red crosses). The circles superimposed with the box plots show the raw data. Statistical significance was tested with a linear regression model (S4 and S5 Figs). * p-value < 0.001.

**Fig 6:**
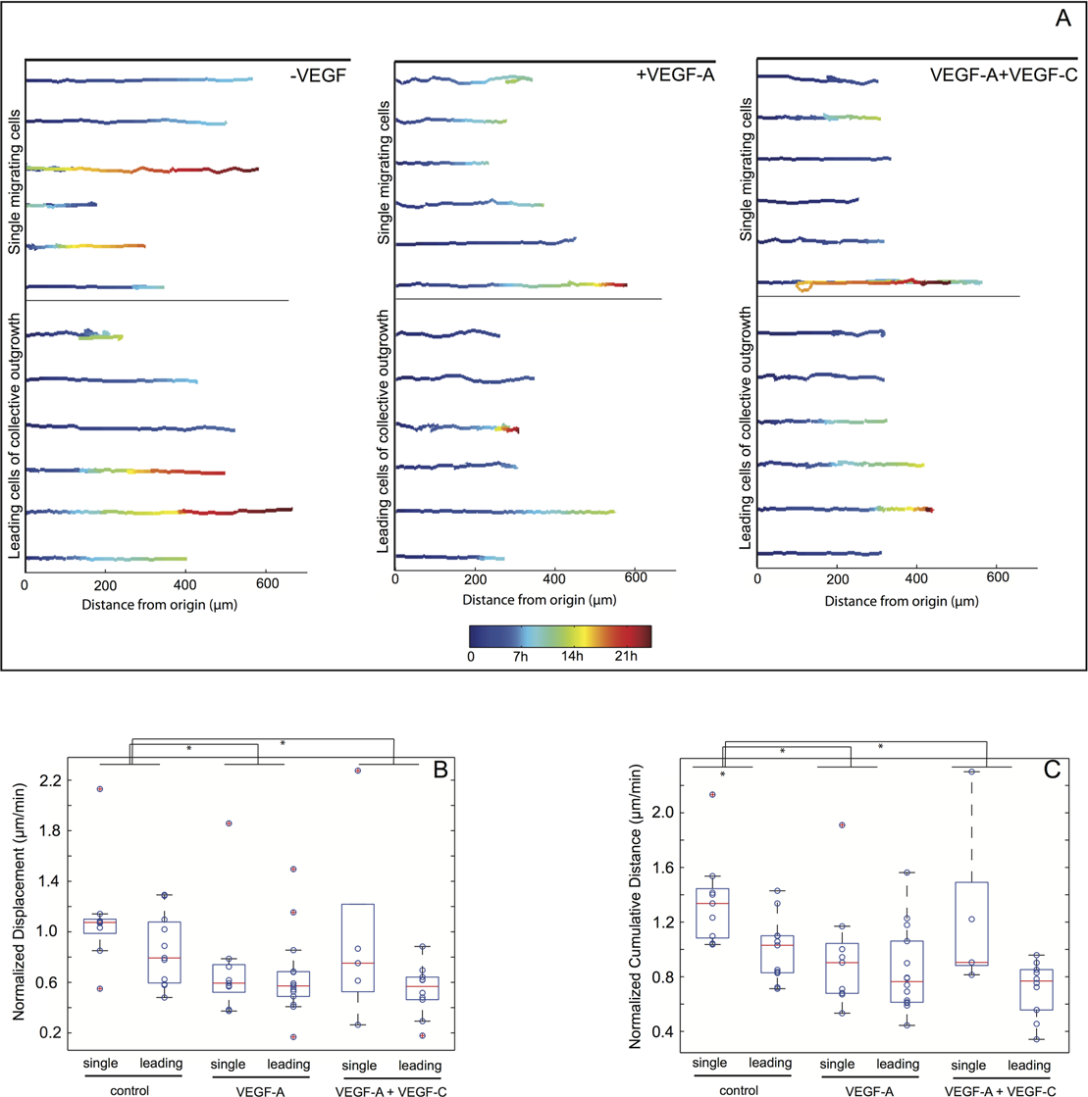
Leading cells of collective HUVEC outgrowth events exhibit higher motion persistence. HUVEC coated beads were incubated with Fn fibers for 6h and then observed with phase contrast video-lapse microscopy. (A) Representative trajectories of cells migrating along Fn fibers, either as single cells (upper panel) or as leading cells of collective outgrowth events (lower panel), in the absence (left) or presence of 50 ng/ml VEGF-A (middle) or 50 ng/ml VEGF-A plus 200 ng/ml VEGF-C (right). The cell tracks shown have been normalized with respect to their origin, as well as the fiber angle. The color denotes time, from the beginning of the track (blue) until 24h (red). From all the cell trajectories analyzed, normalized displacements (B) and cumulative distances (C) travelled by the cells are plotted as box plots, where the central mark is the median (16 to 50 beads per conditions were analyzed), the edges of the box are the 25^th^ and 75^th^ percentiles, the whiskers extend to the most extreme data points not considering outliers (red crosses). The circles superimposed with the box plots show the raw data. Statistical significance was tested with a linear regression model (S7 and S8 Fig). * p-value < 0.001.

Given the length of the manually pulled Fn fibers, our assay is well suited to quantify migration speed and persistence. We thus quantified features of single and collective modes of cell outgrowth by tracking single migrators or the leading cell of a collective unit until they left the field of view, became incorporated into another bead, underwent apoptosis or divided. Representative cell trajectories for single or leading cells are shown (Fig 5A for LEC and Fig 6A for HUVEC), along with two motion parameters: a) displacement (Fig 5B for LEC and Fig 6B for HUVEC), defined as the distance between the original and final position, normalized against the total traveled time, and b) cumulative distance (Fig 5C for LEC and Fig 6C for HUVEC) defined as the sum of the distance travelled for each individual step, also normalized against the total time traveled. The results of this analysis (as described in more detail in the Method Section and in S2–S9 Figs) showed that for LEC, the cumulative distance was significantly lower for the leading cells compared to the single cells, under both control and VEGF-induced conditions, while the differences between the mean displacement values were not statistically significant (Fig 5B and 5C). A higher value of cumulative distance for the same displacement indicates a more frequent switching between forward and backward movement of single cells compared to the movement of collective units, which can also directly be observed in the examples shown in S6 and S7 Videos for LEC and S8 and S9 Videos for HUVEC, suggesting that the movement of leading cells or generally collective cell units is directionally more persistent than that of single cells. This explanation is supported only when the cell velocity does not change, as shown in S6A Fig for 5 representative outgrowth events of each category (control/single, control/collective, VEGF-A/single and VEGF-A/collective). Interestingly, for both the leading cell and single cell categories, the addition of VEGF-A did not have a significant effect on neither displacement nor cumulative distance (Fig 5B and 5C). These results support the hypothesis that VEGF-A regulates LEC migration on Fn fibers by primarily promoting collective cell outgrowth and consequently increasing the persistence of migration directionality without the presence of an explicit biochemical gradient. In contrast to LEC, both displacement and cumulative distance values for HUVEC were decreased in collective outgrowth and also under VEGF-A stimulation, either when VEGF-A was added alone or together with VEGF-C (Fig 6B and 6C), while the profiles of cell velocities (S6B Fig) were very similar across the different conditions, as with LEC. Despite these differences, the HUVEC outgrowth pattern could originate as in the case of LEC, from a higher degree of movement persistence in collective outgrowth or in the presence of VEGF-A. For both LEC and HUVEC and under all conditions tested, the cells were tracked, on average, for a similar amount of time (S9 Fig), ensuring that the observed differences in the migration patterns did not originate from differences in tracking time.

### VE-cadherin is present in cell-cell junctions of collectively outgrowing cells

**Fig 7:**
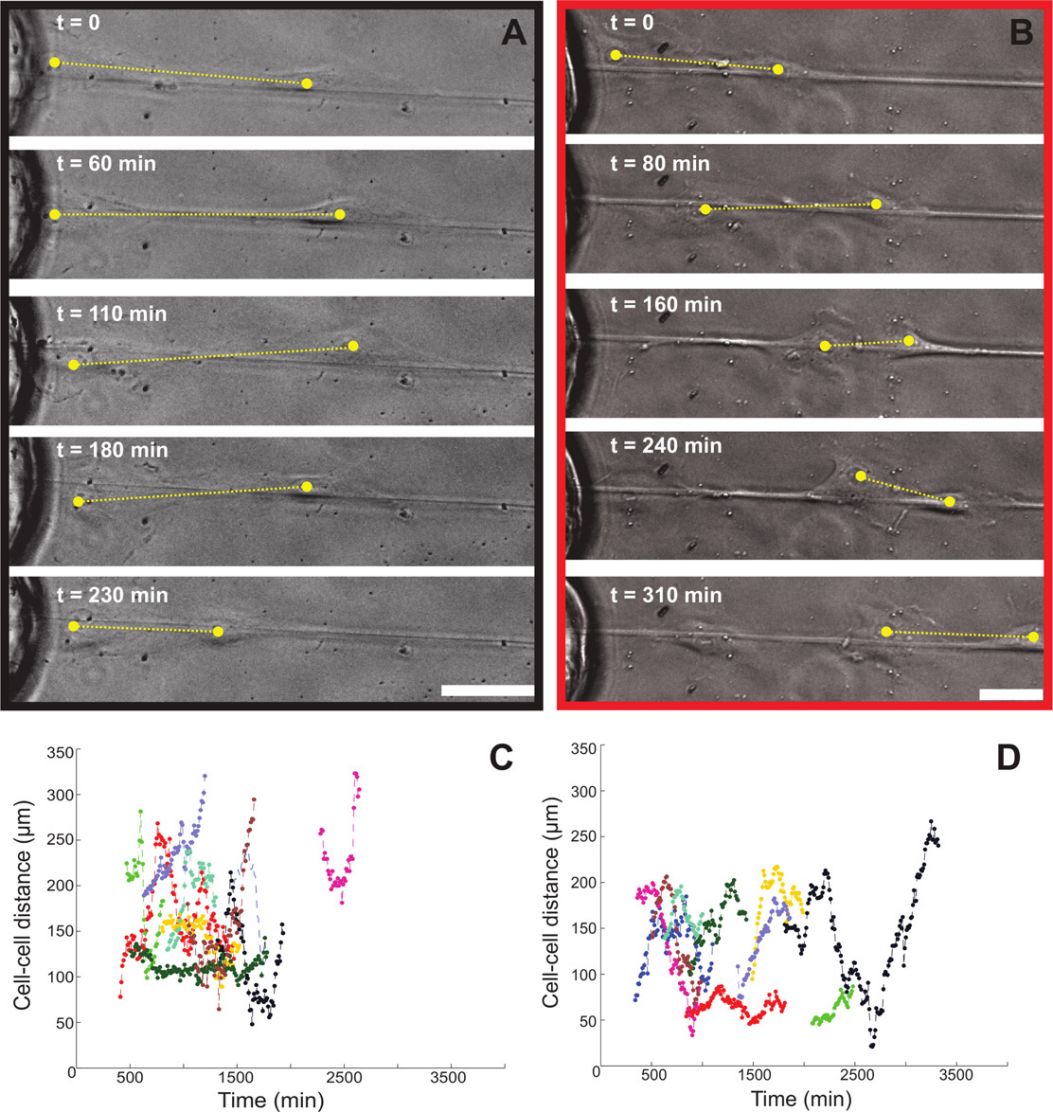
Cell-cell distance does not remain constant during LEC outgrowth. Samples were prepared as described for Fig 4. Cell pairs that moved collectively as a unit were tracked over time and the distance between the cells was measured. (A) A cell pair in the absence of VEGF-A, where the distance between the cells is shortened by the backward movement of the leading cell, whereas the position of the following cell remains mostly constant (S10 Video). (B) A cell pair in the absence of VEGF-A, where the distance between the cells is initially shortened and subsequently lengthened, whereas both cells are moving forward (S11 Video). The distances between 10 cell pairs in the absence (C) or presence (D) of 50 ng/ml soluble VEGF-A in the medium, were plotted over time. Scale bar represents 50 μm.

**Fig 8:**
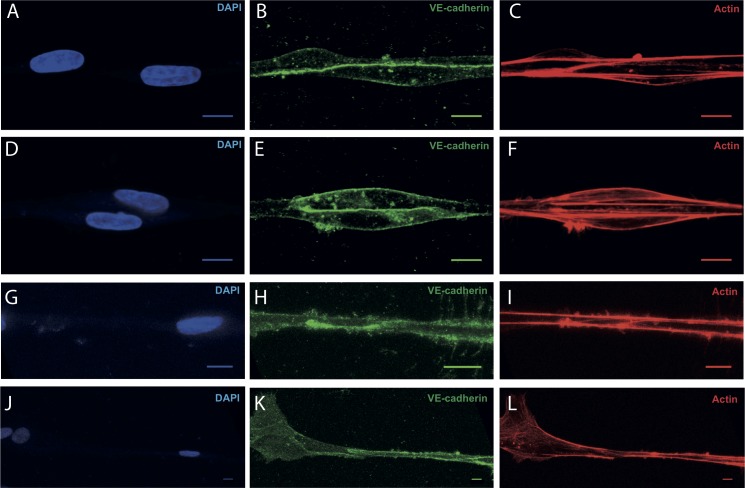
Connections between cells in collective outgrowth events are mediated by VE-cadherin junctions. Samples were prepared as described for Figs 5 and 6, they were fixed with formaldehyde and embedded in collagen gels prior to immunostaining and confocal imaging. HUVEC (A-F) or LEC (G-L) pairs from collective outgrowth events on Fn fibers, in the presence (A-C for HUVEC and G-I for LEC) or absence (D-F for HUVEC and J-L for LEC) of 50 ng/ml VEGF-A are shown stained with DAPI (A, D, G, J), anti-VE-cadherin (B, E, H, K) and phalloidin (C, F, I, L). Scale bar: 10 μm.

Given the capability of our assay to probe single cell behavior during outgrowth to the fibers, we were able to observe the highly dynamic behavior of collective cell outgrowth. For example, the distance between adjacent cells does not remain constant as the collective cell unit migrates along the fiber, as demonstrated by LEC on Fig 7. In the first cell pair shown (Fig 7A and S10 Video), the distance between the cells is occasionally shortened by the backward movements of the leading cell, whereas the position of the cell that followed behind remains more or less constant. In the second case (Fig 7B and S11 Video), the distance between the cells is initially shortened and subsequently lengthened, whereas both cells moved forward. This observation suggests a fine balance between cell-cell and cell-fiber interactions as the collective cell unit migrates along the fiber. With respect to the cell-cell connections, we have identified in several instances of collective outgrowth for both HUVEC and LEC (Fig 8) that cells within a collective unit remain connected via VE-cadherin junctions. In addition, contiguous actin bundles that align with the underlying Fn fiber are observed throughout the cell cluster.

### Active communication between the outgrowing cells and the underlying fibers visualized at the cell-fiber interface by electron microscopy

**Fig 9:**
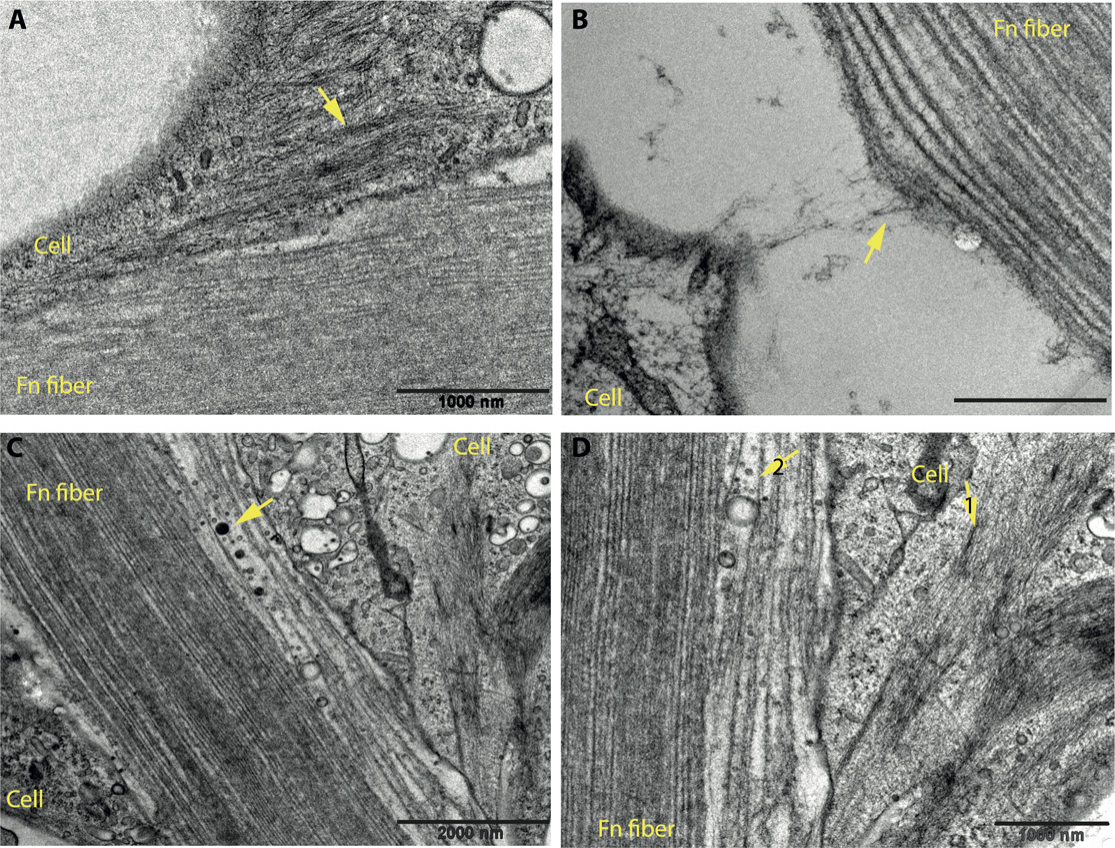
The interface between outgrowing HUVEC and Fn fibers reveals close and active interactions. Samples were prepared as described for Fig 4, they were fixed with formaldehyde and embedded in Epon. 60 nm sections were observed with TEM. Four instances of a cell-fiber interface are shown (A-D). Scale bar: 1000 nm for A, B and D and 2000 nm for C.

The role of the Fn fibers as guiding tracks for endothelial cell outgrowth requires a close and dynamic interaction at the interface between cells and fibers. We investigated this interface by transmission electron microscopy (TEM) of ultrathin sections of our samples. We took advantage of the higher number of outgrowth events observed with HUVEC-coated beads compared to LEC-coated and preformed these experiments with HUVEC. High-pressure frozen samples were Epon embedded and ultrathin sectioned. The sections were observed with TEM (Fig 9). Although, we often observed a significant part of the cell body not in direct contact with the fiber but with the underlying 2D substrate instead, the part of the cell body that comes in contact with the fiber shows a tight interaction; actin fibers are visible in the cell interior and they tend to align with the fiber long axis (Fig 9A, arrow and 9D, arrow 1). We have also observed cell-made fibrils extending from the cell surface to the fiber and pulling on it, starting to separate its internal layers (Fig 9B, arrow). A large number of intracellular vesicles were found at the interface with the fibers, as well as secreted within the fiber (Fig 9C, arrow and 9D, arrow 2), suggesting elevated cellular activity and exploration of the substrate as the cell migrates along the fiber.

## Discussion

Since the importance of Fn for the development and proper functioning of the vasculature is a well-established fact [24–29,40,57], we introduced here a novel fiber-guided (lymph)angiogenesis assay that allows to investigate the role of Fn fibers as guiding tracks for endothelial cell migration during the early stages of (lymph)angiogenesis. A physiological role of fibrillar Fn in regulating angiogenesis has already been suggested from *in vivo* observations that correlate Fn matrix assembly and angiogenic sprouting, as for example in wound healing [58], retinal angiogenesis [25] and embryonic development of major vessel structures [26,29], as well as our findings of colocalization between Fn fibers and lymphatic vessels in the neonatal mouse diaphragm (Fig 1). However, current angiogenesis assays are not specifically designed to dissect the underlying molecular mechanisms of how the fibrillar nature of ECM regulates vessel sprouting. Instead, current *in vivo* assays assess angiogenesis at a tissue or organismic level, integrating many different parameters, and giving a phenotypic output from which it is difficult to identify the contribution of individual factors, especially at the single cell level [59–61]. Similarly, current *in vitro* methods, although probing relatively simple aspects of angiogenesis, such as endothelial cell proliferation, migration or tube formation, are also cell population methods and therefore they also cannot address mechanistic questions at the single cell level [61]. Finally, little is known about the role of fibrillar Fn in the regulation of lymphangiogenesis.

To address these questions, we introduce here an *in vitro* (lymph)angiogenesis assay, where we used microbeads coated with endothelial cells as simple sprouting sources and deposited them on single Fn fibers used as substrates to mimic fibrillar extracellular matrix. The fibers were deposited on a transparent substrate, suitable for live microscopic observation of the ensuing cell outgrowth events at the single cell level. In proof-of-concept studies, this novel experimental setup allowed for several relevant for the field observations.

Fn fibers gave stronger guidance cues for endothelial cell outgrowth as compared to surface-adsorbed Fn (Fig 2), emphasizing the importance of the fibrillar organization of ECM in regulating sprouting. Furthermore, the endothelial cell outgrowth observed was dominated by two types of events: single outgrowth events, where single cells detached from the bead and migrated along the Fn fiber, and collective outgrowth, where a leading cell moved along the Fn fiber followed by a series of trailing cells without disruption of cell-cell contacts (Fig 3 and S1–S3 Videos).

Furthermore, we could identify factors that determine the mode of outgrowth. This is significant in comparison with *in vivo* sprouting where collective outgrowth is important for the early stages of (lymph)angiogenesis, whereby coordinated movement of tip and stalk cells proceeds without the formation of a lumen [16,19]. For LEC, we observe that the addition of VEGF-A in the medium significantly favored collective outgrowth (Fig 4), while it had no effect on either the displacement or cumulative distance traveled by the migrating cells, as determined by individual cell tracking over time (Fig 5). This indicates that soluble VEGF-A does not increase directional movement but rather modulates the quality and coordination of outgrowth events. Although VEGF-A is a well characterized inducer of lymphangiogenesis [6], VEGF-C is considered the typical lymphangiogenic growth factor [7]. Interestingly however, addition of VEGF-C to the medium alone, or in combination with VEGF-A, did not affect the pattern of LEC outgrowth, although, in the concentrations used here, VEGF-C was able to stimulate sprouting when the LEC-coated beads were embedded in a collagen gel [43]. Therefore, it is possible that the output of VEGF-C stimulation depends on the composition and physical properties of the surrounding matrix. Whether this has physiological implications and the response of LEC is different in Fn or collagen rich tissues, requires further investigation. In contrast to LEC, the dynamics of HUVEC outgrowth were different: collective outgrowth was prominent even in the absence of any angiogenic factors and addition of VEGF-A and/or VEGF-C did not change the pattern of outgrowth (Fig 4).

Quantification of the movement of cells within collectively outgrowing cell clusters revealed higher directionality persistence compared to single cell outgrowth for both LEC and HUVEC (Figs 5 and 6). This could originate from the fact that the presence of Fn fibers restricts the dimensionality of cell movement into one dimension, forward and backward, and the presence of trailing cells behind the leading cell makes the backward movement more unfavorable. This observation suggests that purely physical factors provided by the ECM can dictate directed cell motion, even in the absence of explicit chemotactic gradients, adding an additional level of complexity to directional vessel sprouting, not considered before.

In collective outgrowth, the distances between adjacent cells are fluctuating (Fig 7 and S10 and S11 Videos) suggesting that cells pull on each other without loosing contact. Such observations, in conjunction with studies showing that the properties of ECM modulate cell-ECM traction with a direct effect on cell-cell tension [62,63], can be used in the future to advance the mechanistic understanding of the influence of traction forces on directional capillary sprouting. Additionally, they demonstrate a highly dynamic behavior of cell outgrowth that recapitulates another *in vivo* aspect of angiogenic sprouting [19,20]. This behavior has been correlated with differential response to VEGF signaling due to alterations in VE-cadherin mediated cell-cell adhesion [20]. Indeed, cell-cell junctions in our system were also found to be mediated by VE-cadherin (Fig 8). Future work and a promising application of our assay can test whether interaction with Fn is one of the causative factors responsible for such an individualistic response to VEGF during sprouting.

Taken together, our proof-of-concept studies suggest that the collective sprouting and migration of lymphatic and blood endothelial cells along Fn fibers are regulated differently by the presence of VEGF-A or VEGF-C, possibly leading to distinct angiogenic phenotypes. A deeper understanding of such interplay between cell behavior, soluble growth factors and guidance cues from fibrillar ECM can only be gained by exploiting a new generation of (lymph)angiogenesis assays that study sprouting in fibrillar microenvironments. Here we targeted the very first steps of sprouting (lymph)angiogenesis but not questions concerning the later stages, such as lumen formation, that eventually lead to the creation of a functional vascular system [64,65].

The physiological relevance of our fiber-assay is strengthened due to the strong conformational similarity between Fn fibers in the ECM and our manually pulled fibers, as previously established by FRET-based observations [44]. This suggests that the better migratory cues provided by the fibers may be related to Fn conformation, although one cannot exclude the possibility that geometrical and/or Fn density factors may also play a role. Additionally, clustering of extracellular matrix Fn fibrils into thick bundles of a diameter at the μm range can be readily seen in microscopy images of Fn-rich matrices (for an example see [66]). Indeed, in our neonatal mouse diaphragm example (Fig 1), the diameter of the Fn fibers associated with the newly grown lymphathic vessels falls within the μm range. In another example, extracellular matrix Fn fibrils, ranging in diameter from 0.7 to 2.8 μm and in some areas arranged in parallel patterns as well, guide mesoderm migration during gastrulation [67]. The close interaction between the outgrowing cells and the underlying Fn fibers was confirmed by visualization of the cell-fiber interface by electron microscopy (Fig 9).

**Fig 10:**
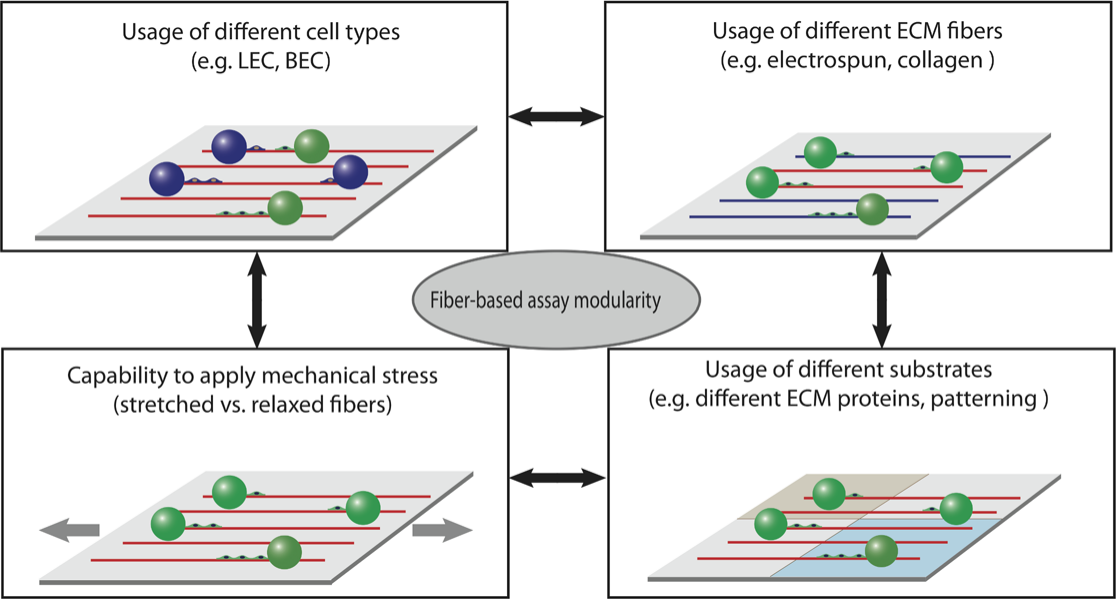
The modularity of the fiber-based assay allows versatile experimental setups. Schematic showing how the three major components of the fiber-guided assay can be modified to address a multitude of questions in the field of (lymph)angiogenesis and beyond: the beads can be cultured with different cell types, different fibers can be used, and the chemistry and/or topography of the underlying substrate can be modified. In addition, the capability to apply mechanical stress allows investigating the role of biomechanical forces in all questions asked.

While we illustrate the power of this new assay using Fn fibers, we want to emphasize its modularity (Fig 10): one can envision the use of other types of fibrils, for example electro-spun nanofibers of collagen, other ECM proteins or even synthetic materials to assess their compatibility and interaction with cellular systems. Moreover, the well established [68] usage of beads in angiogenesis assays was previously extended to other cells in addition to LEC and HUVEC and is also compatible with cancer cell lines [43], thus allowing to address a broader range of questions in vascular biology and beyond.

## Supporting Information

S1 Fig Outgrowth events originating from the same bead are correlated. (A) Schematic representation of multiple outgrowth events from the same bead following a random model. (B) Table summarizing multiple outgrowth events per bead. The number of consecutive events of the same type (single or collective) were counted for all beads that gave rise to multiple outgrowth events during the course of one time-lapse video and the observed frequency of such events was compared to the frequency predicted by a completely random model of outgrowth, such as presented in (A), whereby each outgrowth event has an equal probability to be either single or collective independent of whether the preceding event was single or collective. The statistical significance of the difference between expected and observed frequencies was tested with the chi-square test (chi^2) and the resulting p-values are shown. Beads that gave rise to 2 and 3 outgrowth events were observed for both LEC and HUVEC (LEC: 100 beads with 2 events and 16 beads with 3 events; HUVEC: 78 beads with 2 events and 51 beads with events), whereas beads with more than 3 outgrowth events were observed only for HUVEC (38 beads with 4 events). In all cases and for both LEC and HUVEC, the probability that 2 consecutive outgrowth events from the same bead were of the same type (single or collective) was significantly higher than the probability expected from a completely random model of outgrowth. On the contrary, the probability that 3 consecutive events belonged to the same type was not different than the expected one based on the random model. Curiously, 4 consecutive events showed again higher probability of belonging to the same type than in a random model, but only 10 such beads were counted and a higher number of observations may be needed to accurately assess the probability of such events. These results point to a degree of correlation (although not a 100% correlation) between outgrowth events originating from the same bead, suggesting that the factors determining the mode of outgrowth act at the level of the bead and consequently, a large proportion of the cell population on the bead acts in a similar fashion.
(PDF)

S2 Fig Migration parameters (displacement and cumulative distance) of cells outgrowing from the same bead show stronger correlation compared to migration parameters of cells originating from different beads. Normalized displacement and cumulative distance values (dspl/t and cumD/t respectively as defined for Figs 5 and 6) were calculated from beads with single cell outgrowth events ((A) for LEC and (C) for HUVEC) and beads with collective outgrowth events ((B) for LEC and (D) for HUVEC). The data were pooled across all beads and total average and range values were calculated (range is defined as the difference between the maximum and minimum value of the data population). Bead average and range values were also calculated for the multiple events of each individual bead. In all cases, the ratio of bead over total range was significantly smaller than 1 (t-test p-values: LEC single dspl/t < 0.0001, LEC single cumD/t < 0.0001, LEC collective dspl/t < 0.0001, LEC collective cumD/t = 0.0004, HUVEC single dspl/t = 0.0102, HUVEC single cumD/t = 0.0056, HUVEC collective dspl/t < 0.0001, HUVEC collective cumD/t < 0.0001), showing that parameters for outgrowth events of the same mode (single or collective) that originate from the same bead are systematically smaller that the range of values across different beads and suggesting a degree of correlation between events from the same bead. In contrast to the range values, the ratio of bead to total average was equal to 1 (t-test p-values: LEC single dspl/t = 0.9539, LEC single cumD/t = 0.8948, LEC collective dspl/t = 0.9943, LEC collective cumD/t = 0.9944, HUVEC single dspl/t = 0.8626, HUVEC single cumD/t = 0.8089, HUVEC collective dspl/t = 0.8190, HUVEC collective cumD/t = 0.8587), as expected from data originating from the same population. The data are plotted as box plots, where the central mark is the median, the edges of the box are the 25^th^ and 75^th^ percentiles, the whiskers extend to the most extreme data points not considering outliers (red crosses). The circles superimposed with the box plots show the raw data. The numbers of beads analyzed were 8 for LEC/single, 8 for LEC/collective, 5 for HUVEC/single and 7 for HUVEC/collective. The raw data, as well as the bead and total range and average values for all beads analyzed are summarized in S2 Table.
(PDF)

S3 Fig Normality test of displacement and cumulative distance values. The normalized displacement and cumulative distance values (dspl/t and cumD/t respectively) that were used in Figs 5 and 6 were tested whether they follow a normal distribution. QQ plots plotting the quantiles of the data population (blue corsses) against the quantiles of a normal distribution (red line) are shown: (A) LEC dspl/t, (B) LEC cumD/t, (C) HUVEC dspl/t and (D) HUVEC cumD/t. Data from a population following a normal distribution should fall into the red line. The data from all four populations fit reasonably well to the normal distribution, supporting the usage of statistical tests that assume normality.
(PDF)

S4 Fig Linear regression model of LEC migration parameters against VEGF stimulation. To test whether the presence of VEGF-A had a significant effect on the normalized displacement and cumulative distance values (dspl/t and cumD/t respectively) presented in Fig 5 (LEC) the data were fit with a linear regression model with the response variable being dspl/t or cumD/t and the predictor variable being a categorical variable with two levels: 0 for control and 1 for VEGF-A. The results of the fit (coefficient estimates and statistics for the null hypothesis that the coefficients are zero) are shown in (A) for dspl/t and (B) for cumD/t. In both cases, the p-value is larger than 0.05, showing that neither displacement nor cumulative distance is affected by the presence of VEGF-A. The graphs in (C)-(E) and (F)-(H) show some of the model diagnostics for dspl/t and cumD/t respectively, that validate the linear regression model: (C), (F) Plot of the observation numbers against Cook’s distance, a measure of outliers. If a point exhibits Cook’s distance larger than 1, it needs further investigation as a potential outlier. In this case, no suspect points that need to be excluded from the model can be identified. (D), (G) Lag plot, plotting each residual value against the value of its successive residual. If the errors in the regression are random and independent, as in this case, the scatter of the points in the lag plot should appear random without strong correlations. (E), (H) Normal plot of the ordered regression fit residuals against the corresponding quantiles of the normal distribution. Normally distributed errors and residuals should fall into a straight line, as is here the case. The linear regression was performed in Matlab2014a using a robust regression algorithm, which tolerates outliers and deviations from normality. For a detailed description of the statistics of linear regression and the model diagnostics used in this analysis see ‘Applied Linear Regression’, by S.Weisber (2005; 3d edition).
(PDF)

S5 Fig Linear regression model of LEC migration parameters against outgrowth mode. To test whether the normalized displacement and cumulative distance values (dspl/t and cumD/t respectively) presented in Fig 5 (LEC) are different for single of collective outgrowth the data were fit with a linear regression model with the response variable being dspl/t or cumD/t and the predictor variable being a categorical variable with two levels: 0 for single outgrowth and 1 for collective outgrowth. The analysis was performed the same way as described in S4 Fig. The results of the fit (coefficient estimates and statistics for the null hypothesis that the coefficients are zero) are shown in (A) for dspl/t and (B) for cumD/t. For dspl/t, p-value = 0.208, while for cumD/t, p-value = 0.00094, showing that between single and collective outgrowth only the cumulative distance is different and specifically, smaller than that of single outgrowth, as can be surmised from the negative value of the respective coefficient. The graphs in (C)-(E) and (F)-(H) show some of the model diagnostics for dspl/t and cumD/t respectively, as described in S4 Fig, that validate the linear regression model.
(PDF)

S6 Fig The range of cell velocities is similar for single cells and leading cells of collective outgrowth, in the absence or presence of VEGF-A. Velocities exhibited by outgrowing LEC (A) or HUVEC (B) at each step of their trajectories were plotted for single cells and leading cells of collective outgrowth, in the absence or presence of VEGF-A. The data are plotted as box plots, where the central mark is the median, the edges of the box are the 25^th^ and 75^th^ percentiles, the whiskers extend to the most extreme data points not considering outliers (red crosses). The circles superimposed with the box plots show the raw data. 5 representative cells are shown for each category, each with a different color (blue, red, green, magenta and black).
(PDF)

S7 Fig Linear regression model of HUVEC migration parameters against VEGF-A stimulation. To test whether the normalized displacement and cumulative distance values (dspl/t and cumD/t respectively) presented in Fig 6 (HUVEC) are different in the absence or presence of VEGF-A the data were fit with a linear regression model with the response variable being dspl/t or cumD/t and the predictor variable being a categorical variable with three levels: 0 for control, 1 for VEGF-A and 2 for VEGF-A + VEGF-C. The analysis was performed the same way as described in S4 Fig. The results of the fit (coefficient estimates and statistics for the null hypothesis that the coefficients are zero) are shown in (A) for dspl/t and (B) for cumD/t. For dspl/t, p-value = 0.0017, while for cumD/t, p-value = 0.0019, showing that VEGF-A affects both displacement and cumulative distance values (values are decreased compared to the control as can be surmised by the negative coefficients). The graphs in (C)-(E) and (F)-(H) show some of the model diagnostics for dspl/t and cumD/t respectively, as described in S4 Fig, that validate the linear regression model.
(PDF)

S8 Fig Linear regression model of HUVEC migration parameters against outgrowth mode. To test whether the normalized displacement and cumulative distance values (dspl/t and cumD/t respectively) presented in Fig 6 (HUVEC) are different between single and collective outgrowth the data were fit with a linear regression model with the response variable being dspl/t or cumD/t and the predictor variable being a categorical variable with three levels: 0 for single outgrowth and 1 for collective outgrowth. The analysis was performed the same way as described in S4 Fig. The results of the fit (coefficient estimates and statistics for the null hypothesis that the coefficients are zero) are shown in (A) for dspl/t and (B) for cumD/t. For dspl/t, p-value = 0.259, while for cumD/t, p-value = 0.013, showing that only cumulative distance is different in collective outgrowth and only at the 0.05 level. The graphs in (C)-(E) and (F)-(H) show some of the model diagnostics for dspl/t and cumD/t respectively, as described in S4 Fig, that validate the linear regression model.
(PDF)

S9 Fig Tracking time was similar for single cells and leading cells of collective outgrowth, in the absence or presence of VEGF-A. The total trajectory time of outgrowing cells used for the analysis presented in Fig 5 for LEC (A) and Fig 6 for HUVEC (B) are plotted as box plots, where the central mark is the median, the edges of the box are the 25^th^ and 75^th^ percentiles, the whiskers extend to the most extreme data points not considering outliers (red crosses). The circles superimposed with the box plots show the raw data. For both LEC and HUVEC, there are no statistically significant differences across the 4 conditions (ANOVA p-values: LEC = 0.0999, HUVEC = 0.2757).
(PDF)

S1 TableStatistical analysis of LEC and HUVEC outgrowth activity.A chi-square test was performed to assess whether the percentage of beads that gave rise to single vs. collective outgrowth or to one vs. multiple events per bead were different in the absence or presence of VEGF-A and/or VEGF-C. The observed (obs) numbers of beads under each condition are compared with the expected (exp) numbers, assuming a random distribution across the 4 conditions. For each condition (column) 4 measures are indicated: the chi-square value (chi^2), the residual (res) defined as the difference between observed and expected numbers, the standardized residual (std. res) defined as $\frac{\mathrm{obs}-\exp}{\sqrt{\exp}}$ and the adjusted residual (ads. res) defined as $\frac{\mathrm{obs}-\exp}{\sqrt{\exp*\frac{1-\mathrm{row total}}{n}*\frac{1-\mathrm{column total}}{n}}}$, where n is the number of observations, row total the sum of observation values of the respective row and column total the sum of observation values of the respective column. If the chi-square test shows overall statistical significance, large values of standardized or adjusted residuals (typically larger than 2) point to the groups with the statistical difference. The values of the residuals (res) are not as reliable indicators of statistical significance since they depend on the observed numbers. For a detailed description of chi-square statistical analysis refer to Sharpe D, *Practical Assessment*, *Research and Evaluation*, 2015, 20(8): 1–10.(PDF)

S2 Table Migration parameters of outgrowth events originating from the same bead are more strongly correlated than those of outgrowth events originating from different beads. The raw data of normalized displacement and cumulative distance values of single or collective LEC and HUVEC outgrowth in the absence or presence of VEGF-A and/or VEGF-C used in S2 Fig are shown along with the bead and total range and average values (as defined in S2 Fig).
(PDF)

S1 Video Examples of single and collective LEC outgrowth. Examples of the two classes of LEC outgrowth, originating from the same bead: single cell outgrowth (to the left of the bead) versus collective cell outgrowth (to the right of the bead). Scale bar represents 50 μm and the timer runs in hours:: minutes.
(AVI)

S2 Video Example of single HUVEC outgrowth. Example demonstrating a single HUVEC outgrowth event, originating from one bead. Scale bar represents 50 μm and the timer runs in hours:: minutes.
(AVI)

S3 Video Example of collective HUVEC outgrowth. Example demonstrating a collective HUVEC outgrowth event, originating from one bead. Scale bar represents 50 μm and the timer runs in hours:: minutes.
(AVI)

S4 Video A collective HUVEC outgrowth event, consisting of a large number of individual cells. Scale bar represents 50 μm and the timer runs in hours:: minutes.
(AVI)

S5 Video Several HUVEC single events originating from one bead. Scale bar represents 50 μm and the timer runs in hours:: minutes.
(AVI)

S6 Video Single LEC outgrowth demonstrating frequent changes between forward and backward motion. Scale bar represents 50 μm and the timer runs in hours:: minutes.
(AVI)

S7 Video Collective LEC outgrowth demonstrating persistent motion directionality. Scale bar represents 50 μm and the timer runs in hours:: minutes.
(AVI)

S8 Video Single HUVEC outgrowth demonstrating frequent changes between forward and backward motion. Scale bar represents 50 μm and the timer runs in hours:: minutes.
(AVI)

S9 Video Collective HUVEC outgrowth demonstrating persistent motion directionality. Scale bar represents 50 μm and the timer runs in hours:: minutes.
(AVI)

S10 Video First example of an LEC cell pair migrating collectively while their relative distance changes over time. Scale bar represents 50 μm and the timer runs in hours:: minutes.
(AVI)

S11 Video Second example of an LEC cell pair migrating collectively while their relative distance changes over time. The cell pair depicted in S11 Video belongs to the collective outgrowth event shown also in S1 Video (to the right of the bead). Scale bar represents 50 μm and the timer runs in hours:: minutes.
(AVI)

## Abbreviations

2D: Two-dimensional
3D: Three-dimensional
Dll4: Delta-like ligand 4
ECM: Extracellular matrix
Fn: Fibronectin
HUVEC: Human umbilical vein endothelial cell
LEC: Lymphatic endothelial cell
LYVE-1: Lymphatic vessel hyaluronan receptor-1
VEGF: Vascular endothelial growth factor
VEGFR: Vascular endothelial growth factor receptor

## References

[1] Folkman J (1995) Angiogenesis in cancer, vascular, rheumatoid and other disease. Nat Med 1: 27–31. Available: http://www.ncbi.nlm.nih.gov/pubmed/7584949. 7584949 10.1038/nm0195-27

[2] Potente M, Gerhardt H, Carmeliet P (2011) Basic and therapeutic aspects of angiogenesis. Cell 146: 873–887. 10.1016/j.cell.2011.08.039 21925313

[3] Cueni LN, Detmar M (2008) The lymphatic system in health and disease. Lymphat Res Biol 6: 109–122. Available: http://www.ncbi.nlm.nih.gov/pubmed/19093783. 10.1089/lrb.2008.1008 19093783 PMC3572233

[4] Ferrara N, Gerber HP, LeCouter J (2003) The biology of VEGF and its receptors. Nat Med 9: 669–676. Available: http://www.ncbi.nlm.nih.gov/pubmed/12778165. 12778165 10.1038/nm0603-669

[5] Bruce D, Tan PH (2011) Vascular endothelial growth factor receptors and the therapeutic targeting of angiogenesis in cancer: where do we go from here? Cell Commun Adhes 18: 85–103. Available: http://www.ncbi.nlm.nih.gov/pubmed/22017472. 10.3109/15419061.2011.619673 22017472

[6] Ferrara N (2009) VEGF-A: a critical regulator of blood vessel growth. Eur Cytokine Netw 20: 158–163. Available: http://www.ncbi.nlm.nih.gov/pubmed/20167554. 10.1684/ecn.2009.0170 20167554

[7] Oh SJ, Jeltsch MM, Birkenhäger R, McCarthy JE, Weich HA, Christ B, et al (1997) VEGF and VEGF-C: specific induction of angiogenesis and lymphangiogenesis in the differentiated avian chorioallantoic membrane. Dev Biol 188: 96–109. 10.1006/dbio.1997.8639 9245515

[8] Halin C, Tobler NE, Vigl B, Brown LF, Detmar M (2007) VEGF-A produced by chronically inflamed tissue induces lymphangiogenesis in draining lymph nodes. Blood 110: 3158–3167. Available: http://www.pubmedcentral.nih.gov/articlerender.fcgi?artid=2200913&tool=pmcentrez&rendertype=abstract. 17625067 10.1182/blood-2007-01-066811 PMC2200913

[9] Hirakawa S, Kodama S, Kunstfeld R, Kajiya K, Brown LF, Detmar M (2005) VEGF-A induces tumor and sentinel lymph node lymphangiogenesis and promotes lymphatic metastasis. J Exp Med 201: 1089–1099. Available: http://www.ncbi.nlm.nih.gov/pubmed/15809353. 15809353 10.1084/jem.20041896 PMC2213132

[10] Wuest TR, Carr DJJ (2010) VEGF-A expression by HSV-1-infected cells drives corneal lymphangiogenesis. J Exp Med 207: 101–115. 10.1084/jem.20091385 20026662 PMC2812544

[11] Mallory BP, Mead TJ, Wiginton D a F, Kulkarni RM, Greenberg JM, Akeson AL (2006) Lymphangiogenesis in the developing lung promoted by VEGF-A. Microvasc Res 72: 62–73. Available: http://www.ncbi.nlm.nih.gov/pubmed/16806288. 16806288 10.1016/j.mvr.2006.05.002

[12] Tammela T, Zarkada G, Wallgard E, Murtomäki A, Suchting S, Wirzenius M, et al (2008) Blocking VEGFR-3 suppresses angiogenic sprouting and vascular network formation. Nature 454: 656–660. Available: http://www.ncbi.nlm.nih.gov/pubmed/18594512. 10.1038/nature07083 18594512

[13] Hamada K, Oike Y, Takakura N, Ito Y, Jussila L, Dumont DJ, et al (2000) VEGF-C signaling pathways through VEGFR-2 and VEGFR-3 in vasculoangiogenesis and hematopoiesis. Blood 96: 3793–3800. 11090062

[14] Benest A V, Harper SJ, Herttuala SY, Alitalo K, Bates DO (2008) VEGF-C induced angiogenesis preferentially occurs at a distance from lymphangiogenesis. Cardiovasc Res 78: 315–323. 10.1093/cvr/cvm094 18065770 PMC2613351

[15] Geudens I, Gerhardt H (2011) Coordinating cell behaviour during blood vessel formation. Development 138: 4569–4583. Available: http://www.ncbi.nlm.nih.gov/pubmed/21965610. 10.1242/dev.062323 21965610

[16] Gerhardt H, Golding M, Fruttiger M, Ruhrberg C, Lundkvist A, Abramsson A, et al (2003) VEGF guides angiogenic sprouting utilizing endothelial tip cell filopodia. J Cell Biol 161: 1163–1177. Available: http://www.ncbi.nlm.nih.gov/pubmed/12810700. 12810700 10.1083/jcb.200302047 PMC2172999

[17] Jakobsson L, Franco CA, Bentley K, Collins RT, Ponsioen B, Aspalter IM, et al (2010) Endothelial cells dynamically compete for the tip cell position during angiogenic sprouting. Nat Cell Biol 12: 943–953. Available: http://www.ncbi.nlm.nih.gov/pubmed/20871601. 10.1038/ncb2103 20871601

[18] Zheng W, Tammela T, Yamamoto M, Anisimov A, Holopainen T, Kaijalainen S, et al (2011) Notch restricts lymphatic vessel sprouting induced by vascular endothelial growth factor. Blood 118: 1154–1162. Available: http://www.ncbi.nlm.nih.gov/pubmed/21566091. 10.1182/blood-2010-11-317800 21566091

[19] Arima S, Nishiyama K, Ko T, Arima Y, Hakozaki Y, Sugihara K, et al (2011) Angiogenic morphogenesis driven by dynamic and heterogeneous collective endothelial cell movement. Development 138: 4763–4776. 10.1242/dev.068023 21965612

[20] Bentley K, Franco CA, Philippides A, Blanco R, Dierkes M, Gebala V, et al (2014) The role of differential VE-cadherin dynamics in cell rearrangement during angiogenesis. Nat Cell Biol 16: 309–321. Available: http://www.ncbi.nlm.nih.gov/pubmed/24658686. 10.1038/ncb2926 24658686

[21] Caussinus E, Colombelli J, Affolter M (2008) Tip-cell migration controls stalk-cell intercalation during Drosophila tracheal tube elongation. Curr Biol 18: 1727–1734. Available: http://www.ncbi.nlm.nih.gov/pubmed/19026547. 10.1016/j.cub.2008.10.062 19026547

[22] Friedl P, Gilmour D (2009) Collective cell migration in morphogenesis, regeneration and cancer. Nat Rev Mol Cell Biol 10: 445–457. Available: http://www.ncbi.nlm.nih.gov/entrez/query.fcgi?cmd=Retrieve&db=PubMed&dopt=Citation&list_uids=19546857. 10.1038/nrm2720 19546857

[23] Farhadian F, Contard F, Sabri A, Samuel JL, Rappaport L (1996) Fibronectin and basement membrane in cardiovascular organogenesis and disease pathogenesis. Cardiovasc Res 32: 433–442. Available: http://www.ncbi.nlm.nih.gov/pubmed/8881506. 8881506

[24] Van Obberghen-Schilling E, Tucker RP, Saupe F, Gasser I, Cseh B, Orend G, et al (2011) Fibronectin and tenascin-C: accomplices in vascular morphogenesis during development and tumor growth. Int J Dev Biol 55: 511–525. Available: http://www.ncbi.nlm.nih.gov/pubmed/21769776. 10.1387/ijdb.103243eo 21769776

[25] Stenzel D, Lundkvist A, Sauvaget D, Busse M, Graupera M, van der Flier A, et al (2011) Integrin-dependent and -independent functions of astrocytic fibronectin in retinal angiogenesis. Development 138: 4451–4463. Available: http://www.ncbi.nlm.nih.gov/pubmed/21880786. 10.1242/dev.071381 21880786 PMC3177315

[26] Chiu C-H, Chou C-W, Takada S, Liu Y-W (2012) Development and Fibronectin Signaling Requirements of the Zebrafish Interrenal Vessel. PLoS One 7: e43040 10.1371/journal.pone.0043040 22937010 PMC3428036

[27] George EL, Georges-Labouesse EN, Patel-King RS, Rayburn H, Hynes RO (1993) Defects in mesoderm, neural tube and vascular development in mouse embryos lacking fibronectin. Development 119: 1079–1091. Available: http://www.ncbi.nlm.nih.gov/pubmed/8306876. 8306876 10.1242/dev.119.4.1079

[28] Childs S, Chen J, Garrity DM, Fishman MC (2002) Patterning of angiogenesis in the zebrafish embryo. Development 129: 973–982. 11861480 10.1242/dev.129.4.973

[29] Isogai S, Lawson ND, Torrealday S, Horiguchi M, Weinstein BM (2003) Angiogenic network formation in the developing vertebrate trunk. Development 130: 5281–5290. Available: http://www.ncbi.nlm.nih.gov/pubmed/12954720. 12954720 10.1242/dev.00733

[30] Hagerling R, Pollmann C, Andreas M, Schmidt C, Nurmi H, Adams RH, et al (2013) A novel multistep mechanism for initial lymphangiogenesis in mouse embryos based on ultramicroscopy. EMBO J 32: 629–644. Available: http://www.ncbi.nlm.nih.gov/pubmed/23299940\n http://www.ncbi.nlm.nih.gov/pmc/articles/PMC3590982/pdf/emboj2012340a.pdf. 10.1038/emboj.2012.340 23299940 PMC3590982

[31] Schwarzbauer JE, DeSimone DW (2011) Fibronectins, their fibrillogenesis, and in vivo functions. Cold Spring Harb Perspect Biol 3 Available: http://www.ncbi.nlm.nih.gov/pubmed/21576254. 10.1101/cshperspect.a005041 PMC3119908 21576254

[32] Zamir E, Katz M, Posen Y, Erez N, Yamada KM, Katz BZ, et al (2000) Dynamics and segregation of cell-matrix adhesions in cultured fibroblasts. Nat Cell Biol 2: 191–196. Available: <Go to ISI>://000086397000011. 10783236 10.1038/35008607

[33] Hynes RO (2009) The extracellular matrix: not just pretty fibrils. Science (80-) 326: 1216–1219. Available: http://www.ncbi.nlm.nih.gov/pubmed/19965464. 10.1126/science.1176009 19965464 PMC3536535

[34] Vogel V (2006) Mechanotransduction involving multimodular proteins: converting force into biochemical signals. Annu Rev Biophys Biomol Struct 35: 459–488. Available: http://www.ncbi.nlm.nih.gov/pubmed/16689645. 16689645 10.1146/annurev.biophys.35.040405.102013

[35] Zhou X, Rowe RG, Hiraoka N, George JP, Wirtz D, Mosher DF, et al (2008) Fibronectin fibrillogenesis regulates three-dimensional neovessel formation. Genes Dev 22: 1231–1243. Available: http://genesdev.cshlp.org/content/22/9/1231. 10.1101/gad.1643308 18451110 PMC2335318

[36] Clark RA, Quinn JH, Winn HJ, Lanigan JM, Dellepella P, Colvin RB (1982) Fibronectin is produced by blood vessels in response to injury. J Exp Med 156: 646–651. Available: http://www.ncbi.nlm.nih.gov/pubmed/7047672. 7047672 10.1084/jem.156.2.646 PMC2186773

[37] Clark RA, Lanigan JM, DellaPelle P, Manseau E, Dvorak HF, Colvin RB (1982) Fibronectin and fibrin provide a provisional matrix for epidermal cell migration during wound reepithelialization. J Invest Dermatol 79: 264–269. Available: http://www.ncbi.nlm.nih.gov/pubmed/6752288. 6752288 10.1111/1523-1747.ep12500075

[38] Li J, Zhang YP, Kirsner RS (2003) Angiogenesis in wound repair: angiogenic growth factors and the extracellular matrix. Microsc Res Tech 60: 107–114. Available: http://www.ncbi.nlm.nih.gov/entrez/query.fcgi?cmd=Retrieve&db=PubMed&dopt=Citation&list_uids=12500267. 12500267 10.1002/jemt.10249

[39] Singer AJ, Clark RA (1999) Cutaneous wound healing. N Engl J Med 341: 738–746. Available: http://www.ncbi.nlm.nih.gov/pubmed/10471461. 10471461 10.1056/NEJM199909023411006

[40] Astrof S, Hynes RO (2009) Fibronectins in vascular morphogenesis. Angiogenesis 12: 165–175. Available: http://www.ncbi.nlm.nih.gov/entrez/query.fcgi?cmd=Retrieve&db=PubMed&dopt=Citation&list_uids=19219555. 10.1007/s10456-009-9136-6 19219555 PMC2716138

[41] Hynes RO (2007) Cell-matrix adhesion in vascular development. J Thromb Haemost 5 Suppl 1: 32–40. Available: http://www.ncbi.nlm.nih.gov/pubmed/17635706. 17635706 10.1111/j.1538-7836.2007.02569.x

[42] Mancuso MR, Davis R, Norberg SM, O’Brien S, Sennino B, Nakahara T, et al (2006) Rapid vascular regrowth in tumors after reversal of VEGF inhibition. J Clin Invest 116: 2610–2621. Available: http://www.jci.org/articles/view/24612. 17016557 10.1172/JCI24612 PMC1578604

[43] Schulz MM, Reisen F, Zgraggen S, Fischer S, Yuen D, Kang GJ, et al (2012) Phenotype-based high-content chemical library screening identifies statins as inhibitors of in vivo lymphangiogenesis. Proc Natl Acad Sci U S A 109: E2665–E2674. Available: http://www.ncbi.nlm.nih.gov/pubmed/22949700. 10.1073/pnas.1206036109 22949700 PMC3479568

[44] Little WC, Smith ML, Ebneter U, Vogel V (2008) Assay to mechanically tune and optically probe fibrillar fibronectin conformations from fully relaxed to breakage. Matrix Biol 27: 451–461. Available: http://www.ncbi.nlm.nih.gov/pubmed/18417335. 10.1016/j.matbio.2008.02.003 18417335 PMC5615104

[45] Hirakawa S, Hong YK, Harvey N, Schacht V, Matsuda K, Libermann T, et al (2003) Identification of vascular lineage-specific genes by transcriptional profiling of isolated blood vascular and lymphatic endothelial cells. Am J Pathol 162: 575–586. Available: http://www.ncbi.nlm.nih.gov/pubmed/12547715. 12547715 10.1016/S0002-9440(10)63851-5 PMC1851142

[46] Ejim OS, Blunn GW, Brown RA (1993) Production of artificial-orientated mats and strands from plasma fibronectin: a morphological study. Biomaterials 14: 743–748. Available: http://www.ncbi.nlm.nih.gov/entrez/query.fcgi?cmd=Retrieve&db=PubMed&dopt=Citation&list_uids=8218723. 8218723 10.1016/0142-9612(93)90038-4

[47] Brown R a, Blunn GW, Ejim OS (1994) Preparation of orientated fibrous mats from fibronectin: composition and stability. Biomaterials 15: 457–464. Available: http://www.ncbi.nlm.nih.gov/pubmed/8080937. 8080937 10.1016/0142-9612(94)90225-9

[48] Smith ML, Gourdon D, Little WC, Kubow KE, Eguiluz RA, Luna-Morris S, et al (2007) Force-induced unfolding of fibronectin in the extracellular matrix of living cells. PLoS Biol 5: e268 Available: http://www.ncbi.nlm.nih.gov/pubmed/17914904. 17914904 10.1371/journal.pbio.0050268 PMC1994993

[49] Baneyx G, Baugh L, Vogel V (2002) Fibronectin extension and unfolding within cell matrix fibrils controlled by cytoskeletal tension. Proc Natl Acad Sci U S A 99: 5139–5143. Available: http://www.ncbi.nlm.nih.gov/entrez/query.fcgi?cmd=Retrieve&db=PubMed&dopt=Citation&list_uids=11959962. 11959962 10.1073/pnas.072650799 PMC122735

[50] Kubow KE, Klotzsch E, Smith ML, Gourdon D, Little WC, Vogel V (2009) Crosslinking of cell-derived 3D scaffolds up-regulates the stretching and unfolding of new extracellular matrix assembled by reseeded cells. Integr Biol 1: 635–648. Available: http://www.pubmedcentral.nih.gov/articlerender.fcgi?artid=3818580&tool=pmcentrez&rendertype=abstract. 10.1039/b914996a PMC3818580 20027372

[51] Little WC, Schwartlander R, Smith ML, Gourdon D, Vogel V (2009) Stretched extracellular matrix proteins turn fouling and are functionally rescued by the chaperones albumin and casein. Nano Lett 9: 4158–4167. Available: http://www.ncbi.nlm.nih.gov/entrez/query.fcgi?cmd=Retrieve&db=PubMed&dopt=Citation&list_uids=19743815. 10.1021/nl902365z 19743815 PMC2790870

[52] Chabria M, Hertig S, Smith ML, Vogel V (2010) Stretching fibronectin fibres disrupts binding of bacterial adhesins by physically destroying an epitope. Nat Commun 1: 135 Available: http://www.nature.com/doifinder/10.1038/ncomms1135. 10.1038/ncomms1135 21139580 PMC3105298

[53] Jansen K a, Bacabac RG, Piechocka IK, Koenderink GH (2013) Cells actively stiffen fibrin networks by generating contractile stress. Biophys J 105: 2240–2251. Available: http://www.ncbi.nlm.nih.gov/pubmed/24268136. 10.1016/j.bpj.2013.10.008 24268136 PMC3838739

[54] Yang L, Witten TM, Pidaparti RM (2013) A biomechanical model of wound contraction and scar formation. J Theor Biol 332: 228–248. Available: http://www.ncbi.nlm.nih.gov/pubmed/23563057. 10.1016/j.jtbi.2013.03.013 23563057

[55] Planas-Paz L, Lammert E (2013) Mechanical forces in lymphatic vascular development and disease. Cell Mol Life Sci 70: 4341–4354. Available: http://www.ncbi.nlm.nih.gov/pubmed/23665871. 10.1007/s00018-013-1358-5 23665871 PMC11113353

[56] Hong YK, Lange-Asschenfeldt B, Velasco P, Hirakawa S, Kunstfeld R, Brown LF, et al (2004) VEGF-A promotes tissue repair-associated lymphatic vessel formation via VEGFR-2 and the alpha1beta1 and alpha2beta1 integrins. Faseb J 18: 1111–1113. Available: http://www.ncbi.nlm.nih.gov/pubmed/15132990. 15132990 10.1096/fj.03-1179fje

[57] Chen D, Wang X, Liang D, Gordon J, Mittal A, Manley N, et al (2015) Fibronectin signals through integrin α5β1 to regulate cardiovascular development in a cell type-specific manner. Dev Biol: 1–16. Available: http://linkinghub.elsevier.com/retrieve/pii/S0012160615300658. 10.1016/j.ydbio.2015.09.016 PMC5312697 26434918

[58] Clark RA, DellaPelle P, Manseau E, Lanigan JM, Dvorak HF CR (1982) Blood vessel fibronectin increases in conjunction with endothelial cell proliferation and capillary ingrowth during wound healing. J Invest Dermatol 79: 269–276. Available: http://www.ncbi.nlm.nih.gov/entrez/query.fcgi?cmd=Retrieve&db=PubMed&dopt=Citation&list_uids=6752289. 6752289 10.1111/1523-1747.ep12500076

[59] Malinda KM (2009) In vivo matrigel migration and angiogenesis assay. Methods Mol Biol 467: 287–294. Available: http://www.ncbi.nlm.nih.gov/pubmed/19301678. 10.1007/978-1-59745-241-0_17 19301678

[60] Chen L, Hann B, Wu L (2011) Experimental models to study lymphatic and blood vascular metastasis. J Surg Oncol 103: 475–483. Available: http://www.ncbi.nlm.nih.gov/pubmed/21480239. 10.1002/jso.21794 21480239 PMC3201795

[61] Auerbach R, Lewis R, Shinners B, Kubai L, Akhtar N (2003) Angiogenesis assays: a critical overview. Clin Chem 49: 32–40. Available: http://www.ncbi.nlm.nih.gov/pubmed/12507958. 12507958 10.1373/49.1.32

[62] Maruthamuthu V, Sabass B, Schwarz US, Gardel ML (2011) Cell-ECM traction force modulates endogenous tension at cell-cell contacts. Proc Natl Acad Sci U S A 108: 4708–4713. Available: http://www.ncbi.nlm.nih.gov/pubmed/21383129. 10.1073/pnas.1011123108 21383129 PMC3064395

[63] Korff T, Augustin HG (1999) Tensional forces in fibrillar extracellular matrices control directional capillary sprouting. J Cell Sci 112 (Pt 1: 3249–3258. Available: http://www.ncbi.nlm.nih.gov/pubmed/10504330. 10504330 10.1242/jcs.112.19.3249

[64] Axnick J, Lammert E (2012) Vascular lumen formation. Curr Opin Hematol 19: 192–198. 10.1097/MOH.0b013e3283523ebc 22488306

[65] Sacharidou A, Stratman AN, Davis GE (2012) Molecular Mechanisms Controlling Vascular Lumen Formation in Three-Dimensional Extracellular Matrices. Cells Tissues Organs 195: 122–143. 10.1159/000331410 21997121 PMC3325603

[66] Kubow KE, Vukmirovic R, Zhe L, Klotzsch E, Smith ML, Gourdon D, et al (2015) Mechanical forces regulate the interactions of fibronectin and collagen I in extracellular matrix. Nat Commun 6: 8026 Available: http://www.ncbi.nlm.nih.gov/pubmed/26272817. 10.1038/ncomms9026 26272817 PMC4539566

[67] Sanders E, Hu N, Prasad S (1994) Guidance of filopodial extension by fibronectin-rich extracellular matrix fibrils during avian gastrulation. A study using confocal microscopy. Int J Dev Biol 38: 701–707. Available: http://europepmc.org/abstract/med/7779691. 7779691

[68] Nehls V, Drenckhahn D (1995) A novel, microcarrier-based in vitro assay for rapid and reliable quantification of three-dimensional cell migration and angiogenesis. Microvasc Res 50: 311–322. Available: http://www.ncbi.nlm.nih.gov/pubmed/8583947. 8583947 10.1006/mvre.1995.1061

