# Supplementary material for: Walking the Line: A Fibronectin Fiber-Guided Assay to Probe Early Steps of (Lymph)angiogenesis
Source: PLoS One. 2015 Dec 21;10(12):e0145210. doi: 10.1371/journal.pone.0145210 (PMC4686943; doi:10.1371/journal.pone.0145210)
Supplement: S1 Fig — (A) Schematic representation of multiple outgrowth events from the same bead following a random model. (B) Table summarizing multiple outgrowth events per bead. The number of consecutive events of the same type (single or collective) were counted for all beads that gave rise to multiple outgrowth events during the course of one time-lapse video and the observed frequency of such events was compared to the frequency predicted by a completely random model of outgrowth, such as presented in (A), whereby each outgrowth event has an equal probability to be either single or collective independent of whether the preceding event was single or collective. The statistical significance of the difference between expected and observed frequencies was tested with the chi-square test (chi^2) and the resulting p-values are shown. Beads that gave rise to 2 and 3 outgrowth events were observed for both LEC and HUVEC (LEC: 100 beads with 2 events and 16 beads with 3 events; HUVEC: 78 beads with 2 events and 51 beads with events), whereas beads with more than 3 outgrowth events were observed only for HUVEC (38 beads with 4 events). In all cases and for both LEC and HUVEC, the probability that 2 consecutive outgrowth events from the same bead were of the same type (single or collective) was significantly higher than the probability expected from a completely random model of outgrowth. On the contrary, the probability that 3 consecutive events belonged to the same type was not different than the expected one based on the random model. Curiously, 4 consecutive events showed again higher probability of belonging to the same type than in a random model, but only 10 such beads were counted and a higher number of observations may be needed to accurately assess the probability of such events. These results point to a degree of correlation (although not a 100% correlation) between outgrowth events originating from the same bead, suggesting that the factors determining the mode of outgrowth act [file pone.0145210.s001.pdf]

suppl. Figure 1

A

Event tree for multiple outgrowth events coming from one bead

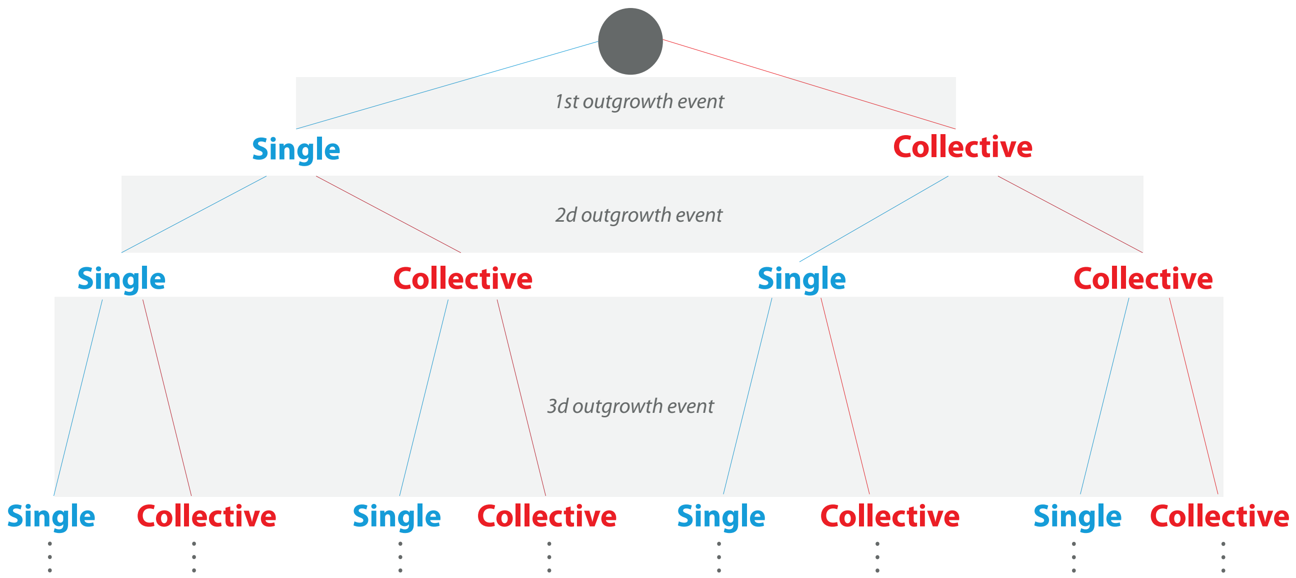

B

Total number of events per bead	Number of consecutive events of the same mode	Expected frequencies (random model)	Observed frequencies (LEC)		Observed frequencies (HUVEC)	
			% of beads with consecutive events	p value (chi^2 obs vs. exp)	% of beads with consecutive events	p value (chi^2 obs vs. exp)
2	2	50	70	0.0039	74	0.0017
3	2	75	100	0.033	100	0.0001
	3	25	50	0.24	37	0.20
4	2	88	-	-	100	0.021
	3	75	-	-	89	0.086
	4	12	-	-	26	<0.00001
