# Supplementary figures and images for: Walking the Line: A Fibronectin Fiber-Guided Assay to Probe Early Steps of (Lymph)angiogenesis
Source: PLoS One. 2015 Dec 21;10(12):e0145210. doi: 10.1371/journal.pone.0145210 (PMC4686943; doi:10.1371/journal.pone.0145210)

suppl. Figure 2

**A**

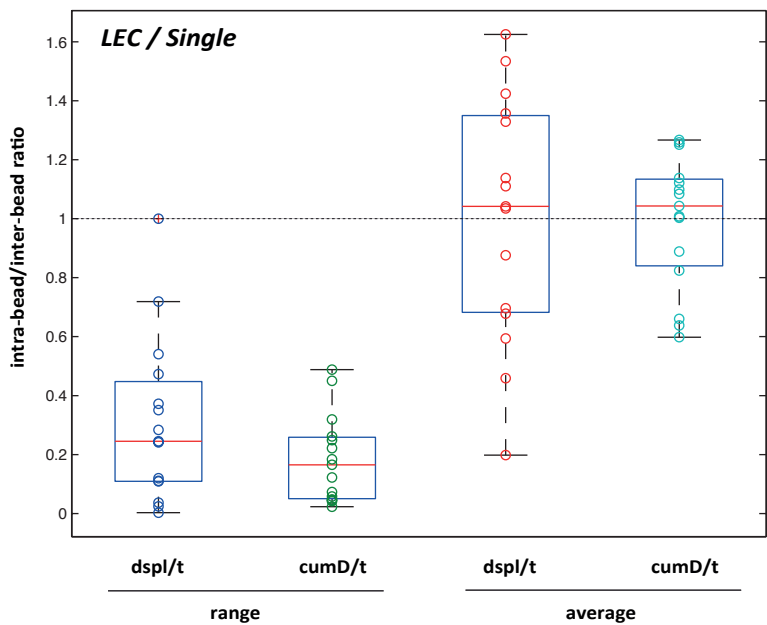

**B**

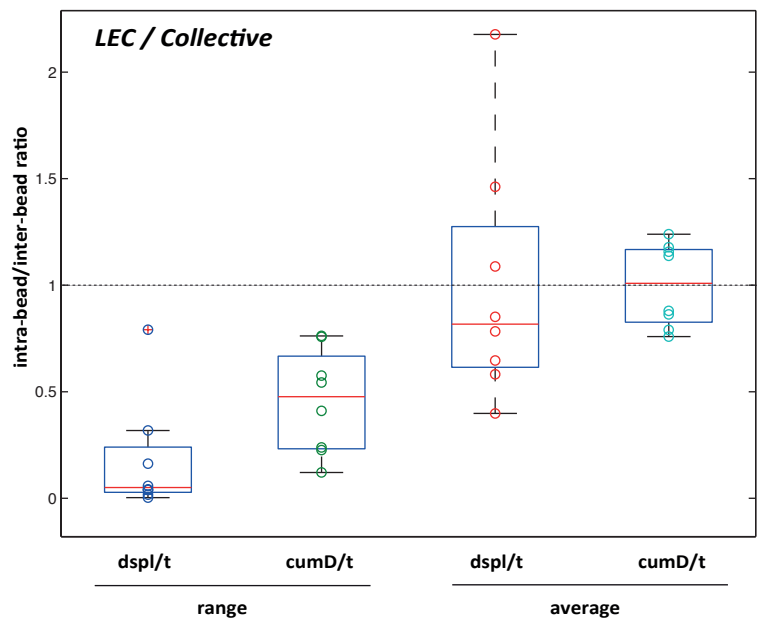

**C**

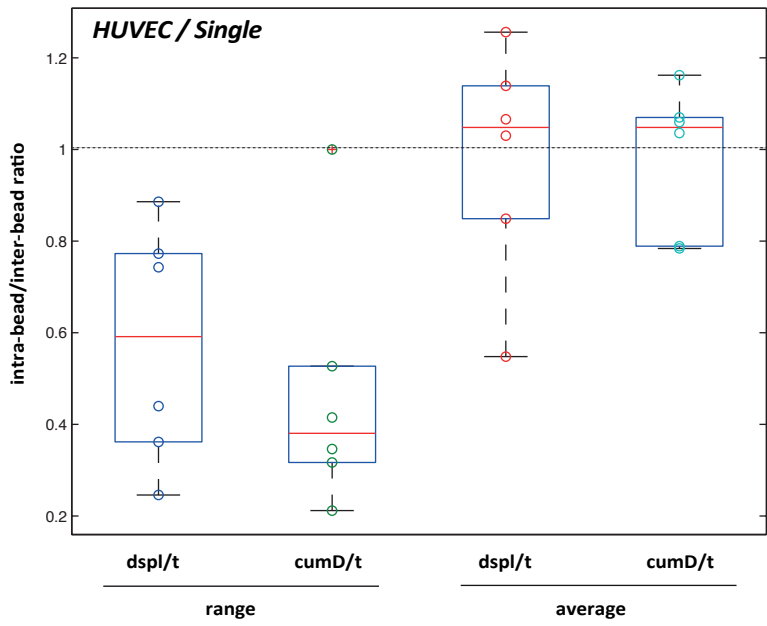

**D**

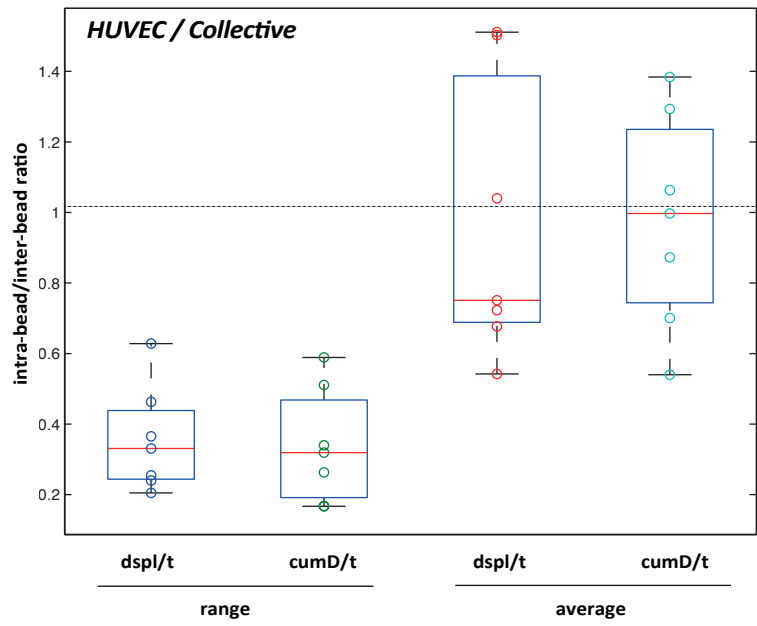

Supplement: S2 Fig — Normalized displacement and cumulative distance values (dspl/t and cumD/t respectively as defined for Figs 5 and 6) were calculated from beads with single cell outgrowth events ((A) for LEC and (C) for HUVEC) and beads with collective outgrowth events ((B) for LEC and (D) for HUVEC). The data were pooled across all beads and total average and range values were calculated (range is defined as the difference between the maximum and minimum value of the data population). Bead average and range values were also calculated for the multiple events of each individual bead. In all cases, the ratio of bead over total range was significantly smaller than 1 (t-test p-values: LEC single dspl/t < 0.0001, LEC single cumD/t < 0.0001, LEC collective dspl/t < 0.0001, LEC collective cumD/t = 0.0004, HUVEC single dspl/t = 0.0102, HUVEC single cumD/t = 0.0056, HUVEC collective dspl/t < 0.0001, HUVEC collective cumD/t < 0.0001), showing that parameters for outgrowth events of the same mode (single or collective) that originate from the same bead are systematically smaller that the range of values across different beads and suggesting a degree of correlation between events from the same bead. In contrast to the range values, the ratio of bead to total average was equal to 1 (t-test p-values: LEC single dspl/t = 0.9539, LEC single cumD/t = 0.8948, LEC collective dspl/t = 0.9943, LEC collective cumD/t = 0.9944, HUVEC single dspl/t = 0.8626, HUVEC single cumD/t = 0.8089, HUVEC collective dspl/t = 0.8190, HUVEC collective cumD/t = 0.8587), as expected from data originating from the same population. The data are plotted as box plots, where the central mark is the median, the edges of the box are the 25th and 75th percentiles, the whiskers extend to the most extreme data points not considering outliers (red crosses). The circles superimposed with the box plots show the raw data. The numbers of beads analyzed were 8 for LEC/single, 8 for LEC/collective, 5 for HUVEC/single and 7 for HUVEC/collec [file pone.0145210.s002.pdf]

suppl. Figure 3

A

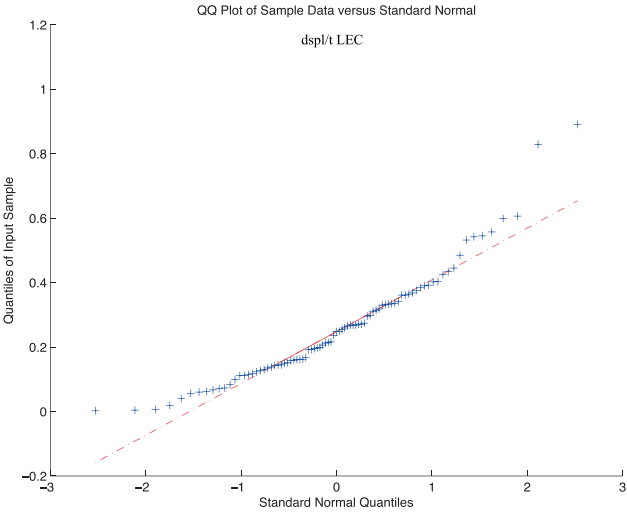

B

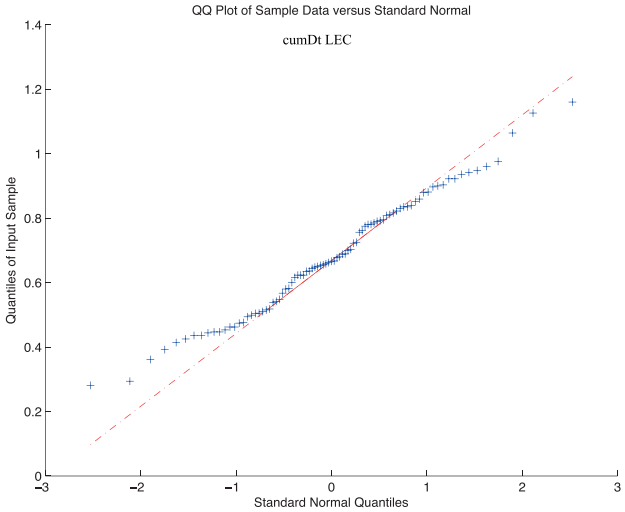

C

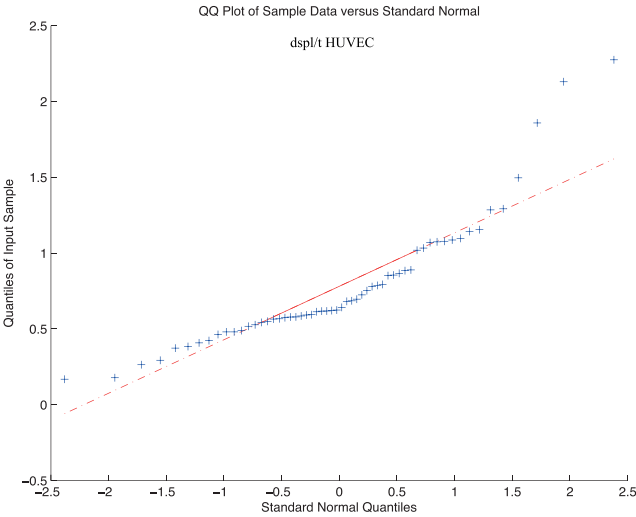

D

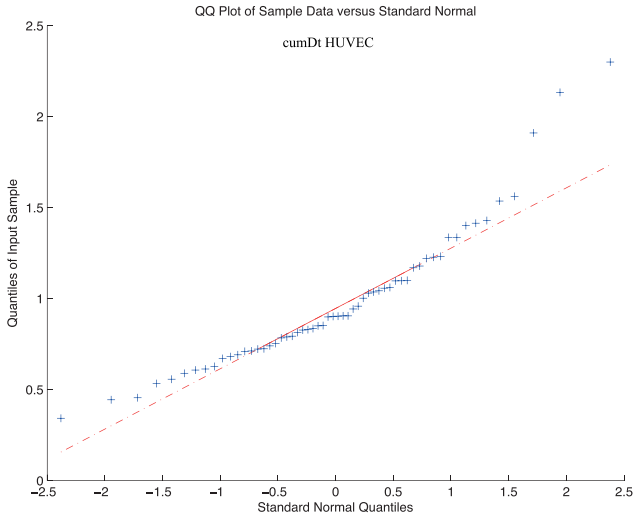

Supplement: S3 Fig — The normalized displacement and cumulative distance values (dspl/t and cumD/t respectively) that were used in Figs 5 and 6 were tested whether they follow a normal distribution. QQ plots plotting the quantiles of the data population (blue corsses) against the quantiles of a normal distribution (red line) are shown: (A) LEC dspl/t, (B) LEC cumD/t, (C) HUVEC dspl/t and (D) HUVEC cumD/t. Data from a population following a normal distribution should fall into the red line. The data from all four populations fit reasonably well to the normal distribution, supporting the usage of statistical tests that assume normality. (PDF) [file pone.0145210.s003.pdf]

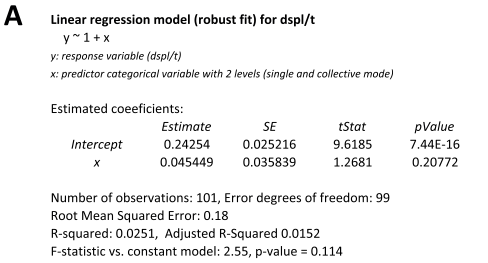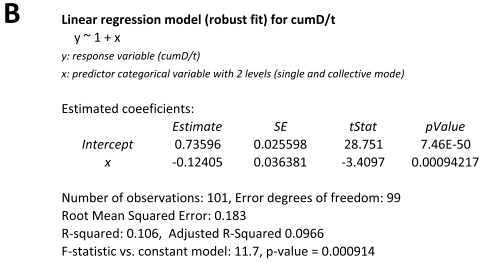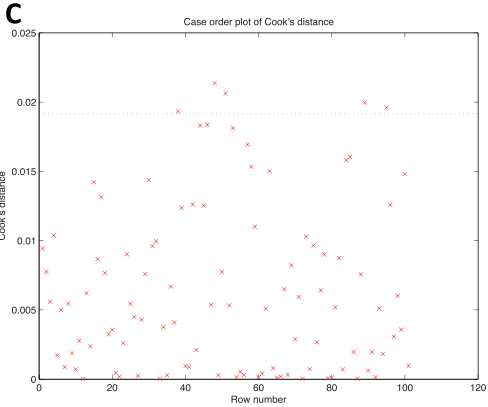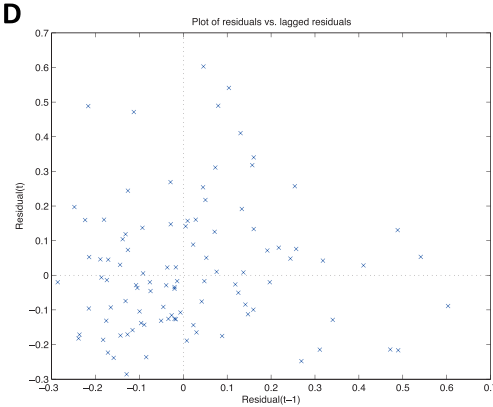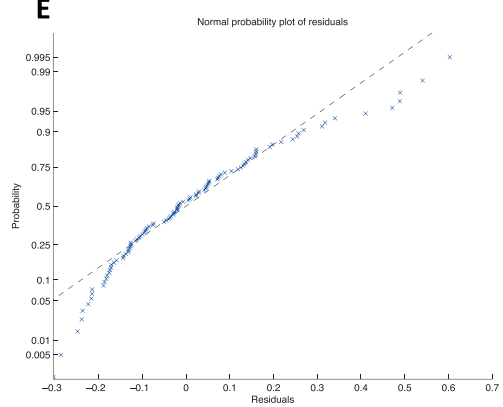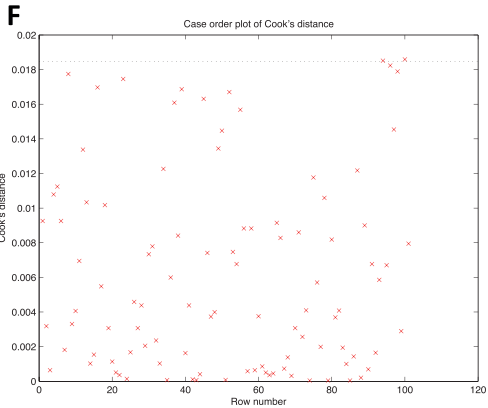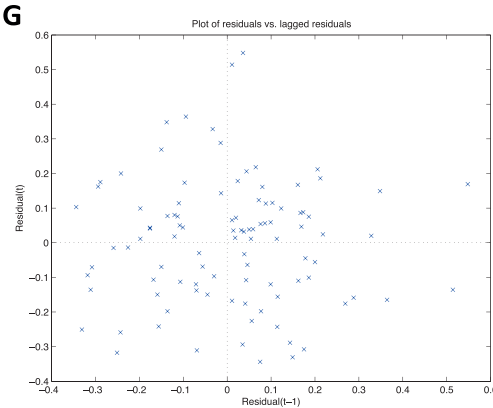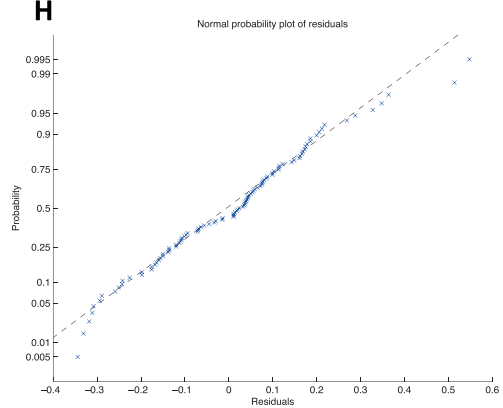

Supplement: S5 Fig — To test whether the normalized displacement and cumulative distance values (dspl/t and cumD/t respectively) presented in Fig 5 (LEC) are different for single of collective outgrowth the data were fit with a linear regression model with the response variable being dspl/t or cumD/t and the predictor variable being a categorical variable with two levels: 0 for single outgrowth and 1 for collective outgrowth. The analysis was performed the same way as described in S4 Fig. The results of the fit (coefficient estimates and statistics for the null hypothesis that the coefficients are zero) are shown in (A) for dspl/t and (B) for cumD/t. For dspl/t, p-value = 0.208, while for cumD/t, p-value = 0.00094, showing that between single and collective outgrowth only the cumulative distance is different and specifically, smaller than that of single outgrowth, as can be surmised from the negative value of the respective coefficient. The graphs in (C)-(E) and (F)-(H) show some of the model diagnostics for dspl/t and cumD/t respectively, as described in S4 Fig, that validate the linear regression model. (PDF) [file pone.0145210.s005.pdf]

suppl. Figure 6

A

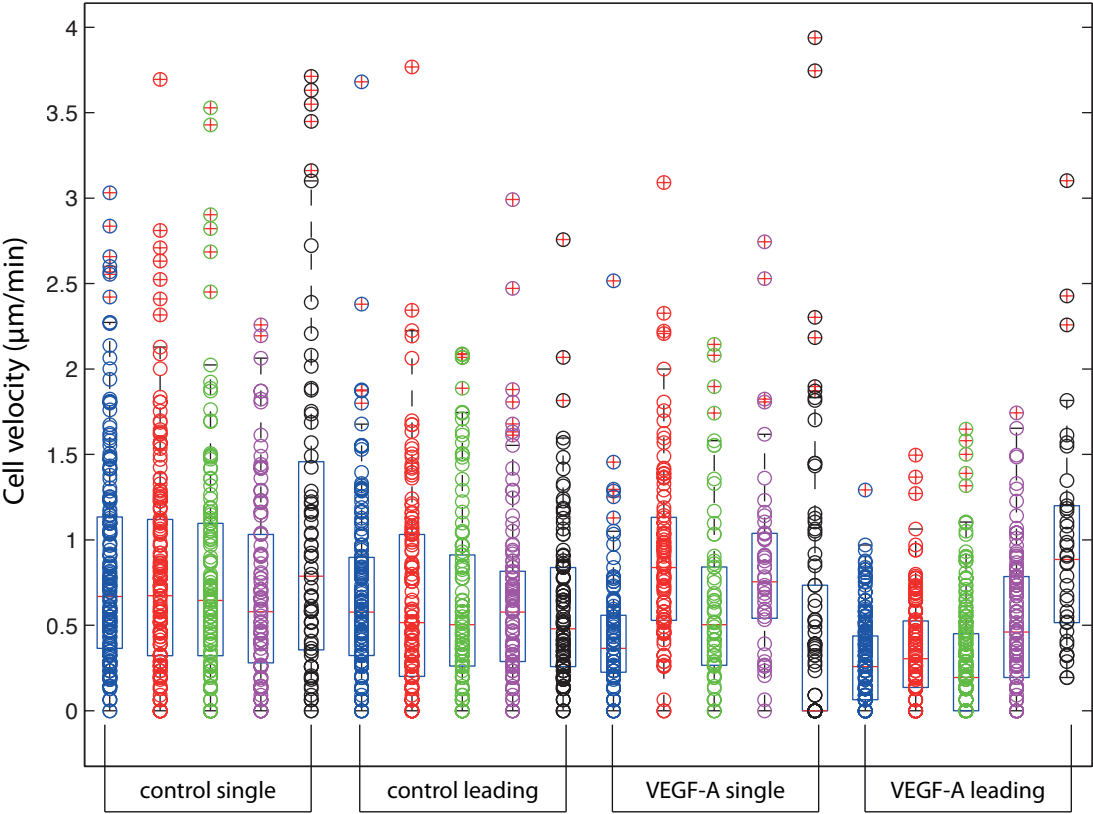

B

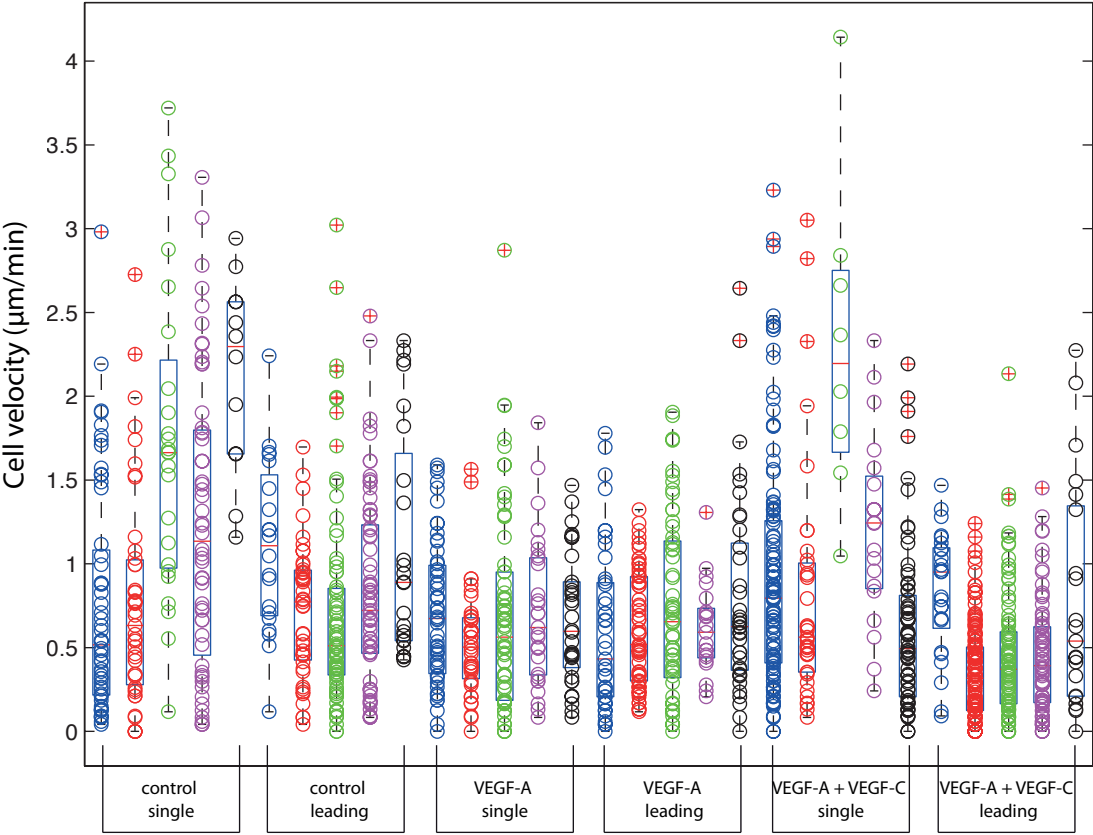

Supplement: S6 Fig — Velocities exhibited by outgrowing LEC (A) or HUVEC (B) at each step of their trajectories were plotted for single cells and leading cells of collective outgrowth, in the absence or presence of VEGF-A. The data are plotted as box plots, where the central mark is the median, the edges of the box are the 25th and 75th percentiles, the whiskers extend to the most extreme data points not considering outliers (red crosses). The circles superimposed with the box plots show the raw data. 5 representative cells are shown for each category, each with a different color (blue, red, green, magenta and black). (PDF) [file pone.0145210.s006.pdf]

suppl. Figure 9

**A**

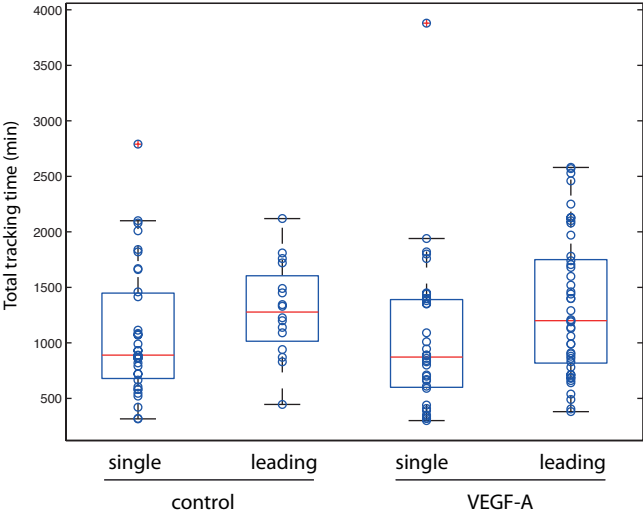

**B**

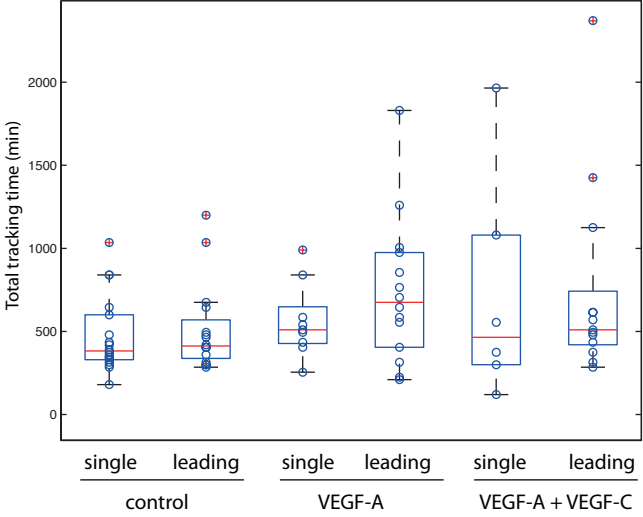

Supplement: S9 Fig — The total trajectory time of outgrowing cells used for the analysis presented in Fig 5 for LEC (A) and Fig 6 for HUVEC (B) are plotted as box plots, where the central mark is the median, the edges of the box are the 25th and 75th percentiles, the whiskers extend to the most extreme data points not considering outliers (red crosses). The circles superimposed with the box plots show the raw data. For both LEC and HUVEC, there are no statistically significant differences across the 4 conditions (ANOVA p-values: LEC = 0.0999, HUVEC = 0.2757). (PDF) [file pone.0145210.s009.pdf]
