# Supplementary material for: Walking the Line: A Fibronectin Fiber-Guided Assay to Probe Early Steps of (Lymph)angiogenesis
Source: PLoS One. 2015 Dec 21;10(12):e0145210. doi: 10.1371/journal.pone.0145210 (PMC4686943; doi:10.1371/journal.pone.0145210)

suppl. Figure 4

A

Linear regression model (robust fit) for dspl/t

y ~ 1 + x

y: response variable (dspl/t)

x: predictor categorical variable with 2 levels (+/- VEGF-A)

Estimated coeeficients:

	Estimate	*SE*	*tStat*	*pValue*
Intercept	0.22978	0.025883	8.8774	9.57E-14
x	0.022839	0.034489	0.66222	0.50962

Number of observations: 87, Error degrees of freedom: 85
Root Mean Squared Error: 0.16
R-squared: 0.0173, Adjusted R-Squared 0.00572
F-statistic vs. constant model: 1.49, p-value = 0.225

B

Linear regression model (robust fit) for cumD/t

y ~ 1 + x

y: response variable (cumD/t)

x: predictor categorical variable with 2 levels (+/- VEGF-A)

Estimated coeeficients:

	Estimate	*SE*	*tStat*	*pValue*
Intercept	0.70264	0.032231	21.8	1.46E-36
x	-0.044551	0.042947	-1.374	0.30251

Number of observations: 87, Error degrees of freedom: 85
Root Mean Squared Error: 0.199
R-squared: 0.0127, Adjusted R-Squared 0.00108
F-statistic vs. constant model: 1.09, p-value = 0.299

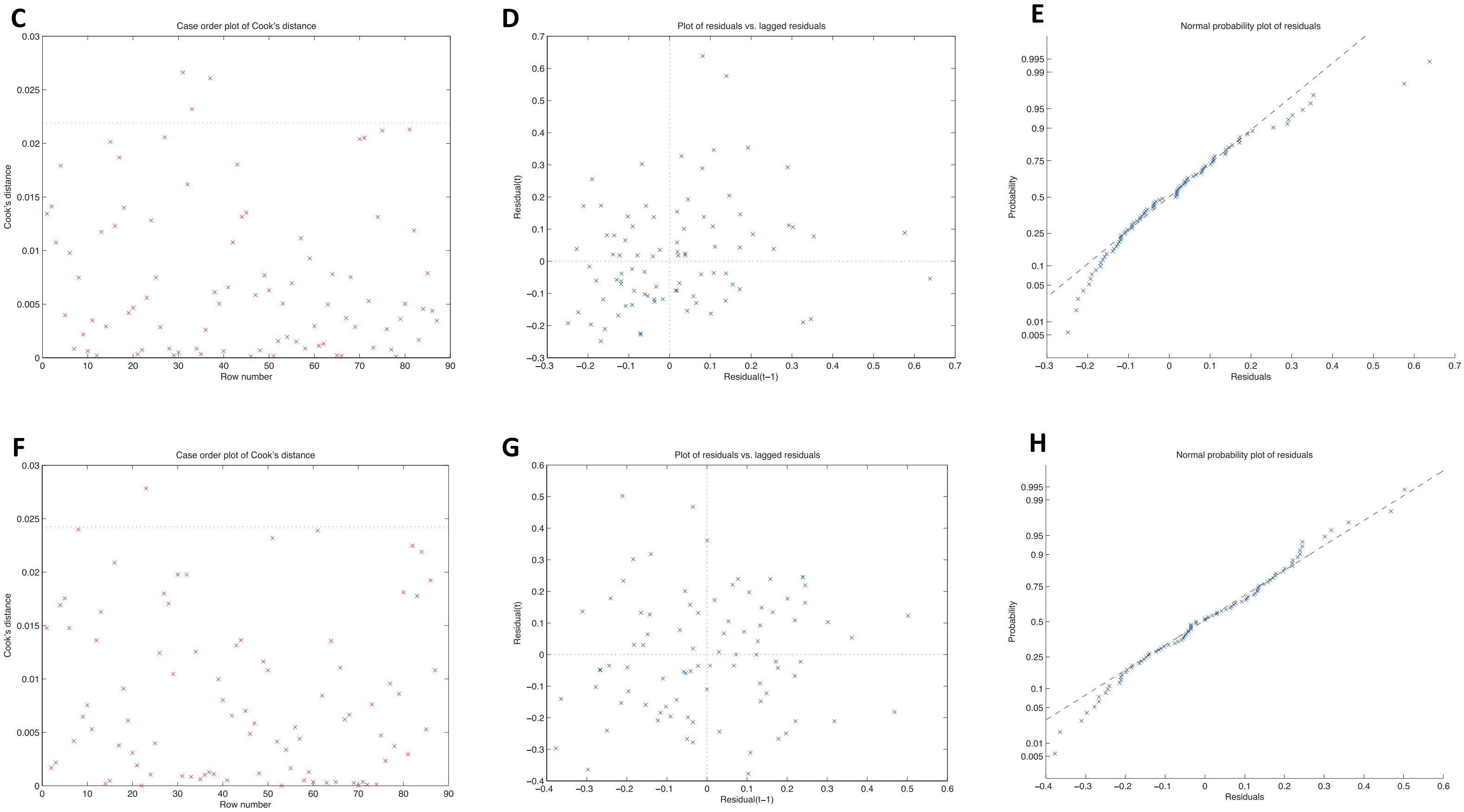

Supplement: S4 Fig — To test whether the presence of VEGF-A had a significant effect on the normalized displacement and cumulative distance values (dspl/t and cumD/t respectively) presented in Fig 5 (LEC) the data were fit with a linear regression model with the response variable being dspl/t or cumD/t and the predictor variable being a categorical variable with two levels: 0 for control and 1 for VEGF-A. The results of the fit (coefficient estimates and statistics for the null hypothesis that the coefficients are zero) are shown in (A) for dspl/t and (B) for cumD/t. In both cases, the p-value is larger than 0.05, showing that neither displacement nor cumulative distance is affected by the presence of VEGF-A. The graphs in (C)-(E) and (F)-(H) show some of the model diagnostics for dspl/t and cumD/t respectively, that validate the linear regression model: (C), (F) Plot of the observation numbers against Cook’s distance, a measure of outliers. If a point exhibits Cook’s distance larger than 1, it needs further investigation as a potential outlier. In this case, no suspect points that need to be excluded from the model can be identified. (D), (G) Lag plot, plotting each residual value against the value of its successive residual. If the errors in the regression are random and independent, as in this case, the scatter of the points in the lag plot should appear random without strong correlations. (E), (H) Normal plot of the ordered regression fit residuals against the corresponding quantiles of the normal distribution. Normally distributed errors and residuals should fall into a straight line, as is here the case. The linear regression was performed in Matlab2014a using a robust regression algorithm, which tolerates outliers and deviations from normality. For a detailed description of the statistics of linear regression and the model diagnostics used in this analysis see ‘Applied Linear Regression’, by S.Weisber (2005; 3d edition). (PDF) [file pone.0145210.s004.pdf]
