# Supplementary material for: Walking the Line: A Fibronectin Fiber-Guided Assay to Probe Early Steps of (Lymph)angiogenesis
Source: PLoS One. 2015 Dec 21;10(12):e0145210. doi: 10.1371/journal.pone.0145210 (PMC4686943; doi:10.1371/journal.pone.0145210)

suppl. Figure 7

A

Linear regression model (robust fit) for dspl/t

y ~ 1 + x

y: response variable (dspl/t)

x: predictor categorical variable with 3 levels (control, VEGF-A and VEGF-A+VEGF-C)

Estimated coeeficients:

	Estimate	*SE*	*tStat*	*pValue*
Intercept	0.91951	0.061376	14.982	4.59E-21
x	-0.33827	0.08392	-4.0308	0.00017248

Number of observations: 58, Error degrees of freedom: 55
Root Mean Squared Error: 0.274
R-squared: 0.322, Adjusted R-Squared 0.298
F-statistic vs. constant model: 13.1, p-value = 2.26e-05

B

Linear regression model (robust fit) for cumD/t

y ~ 1 + x

y: response variable (cumD/t)

x: predictor categorical variable with 3 levels (control, VEGF-A and VEGF-A+VEGF-C)

Estimated coeeficients:

	Estimate	*SE*	*tStat*	*pValue*
Intercept	1.1263	0.066193	17.016	1.42E-23
x	-0.29446	0.090506	-3.2535	0.0019514

Number of observations: 58, Error degrees of freedom: 55
Root Mean Squared Error: 0.296
R-squared: 0.235, Adjusted R-Squared 0.207
F-statistic vs. constant model: 8.46, p-value = 0.000626

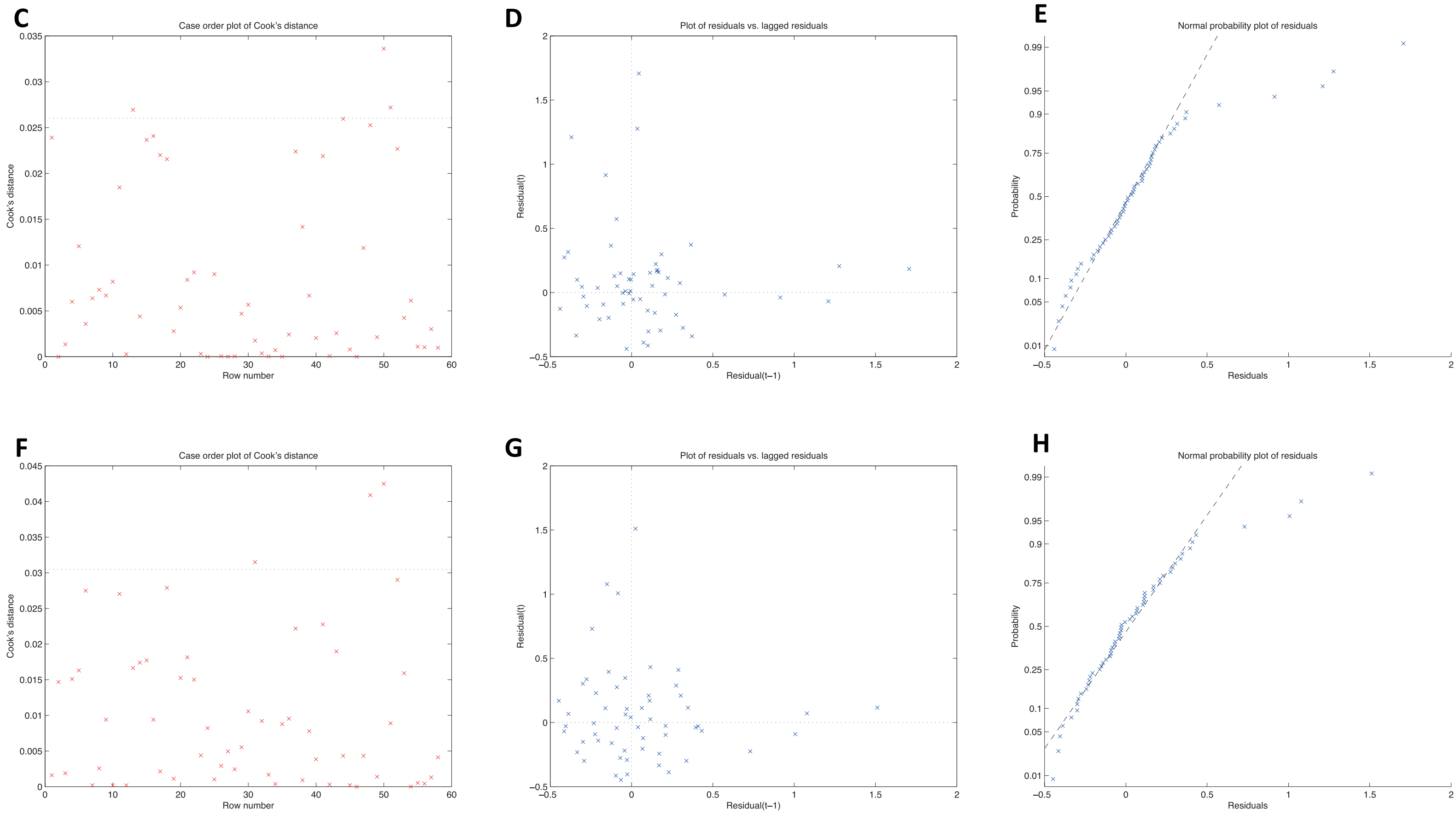

Supplement: S7 Fig — To test whether the normalized displacement and cumulative distance values (dspl/t and cumD/t respectively) presented in Fig 6 (HUVEC) are different in the absence or presence of VEGF-A the data were fit with a linear regression model with the response variable being dspl/t or cumD/t and the predictor variable being a categorical variable with three levels: 0 for control, 1 for VEGF-A and 2 for VEGF-A + VEGF-C. The analysis was performed the same way as described in S4 Fig. The results of the fit (coefficient estimates and statistics for the null hypothesis that the coefficients are zero) are shown in (A) for dspl/t and (B) for cumD/t. For dspl/t, p-value = 0.0017, while for cumD/t, p-value = 0.0019, showing that VEGF-A affects both displacement and cumulative distance values (values are decreased compared to the control as can be surmised by the negative coefficients). The graphs in (C)-(E) and (F)-(H) show some of the model diagnostics for dspl/t and cumD/t respectively, as described in S4 Fig, that validate the linear regression model. (PDF) [file pone.0145210.s007.pdf]
