# Supplementary material for: Walking the Line: A Fibronectin Fiber-Guided Assay to Probe Early Steps of (Lymph)angiogenesis
Source: PLoS One. 2015 Dec 21;10(12):e0145210. doi: 10.1371/journal.pone.0145210 (PMC4686943; doi:10.1371/journal.pone.0145210)

suppl. Figure 8

A

Linear regression model (robust fit) for dspl/t

y ~ 1 + x

y: response variable (dspl/t)

x: predictor categorical variable with 2 levels (single and collective)

Estimated coeeficients:

	Estimate	*SE*	*tStat*	*pValue*
Intercept	0.7591	0.068388	11.1	7.15E-16
x	-0.10105	0.088792	-1.138	0.25988

Number of observations: 59, Error degrees of freedom: 57
Root Mean Squared Error: 0.335
R-squared: 0.076, Adjusted R-Squared 0.0598
F-statistic vs. constant model: 4.69, p-value = 0.0345

B

Linear regression model (robust fit) for cumD/t

y ~ 1 + x

y: response variable (cumD/t)

x: predictor categorical variable with 2 levels (single and collective)

Estimated coeeficients:

	Estimate	*SE*	*tStat*	*pValue*
Intercept	1.0665	0.066891	15.944	1.14E-22
x	-0.22163	0.086848	-2.5519	0.01342

Number of observations: 59, Error degrees of freedom: 57
Root Mean Squared Error: 0.328
R-squared: 0.117, Adjusted R-Squared 0.102
F-statistic vs. constant model: 7.58, p-value = 0.00789

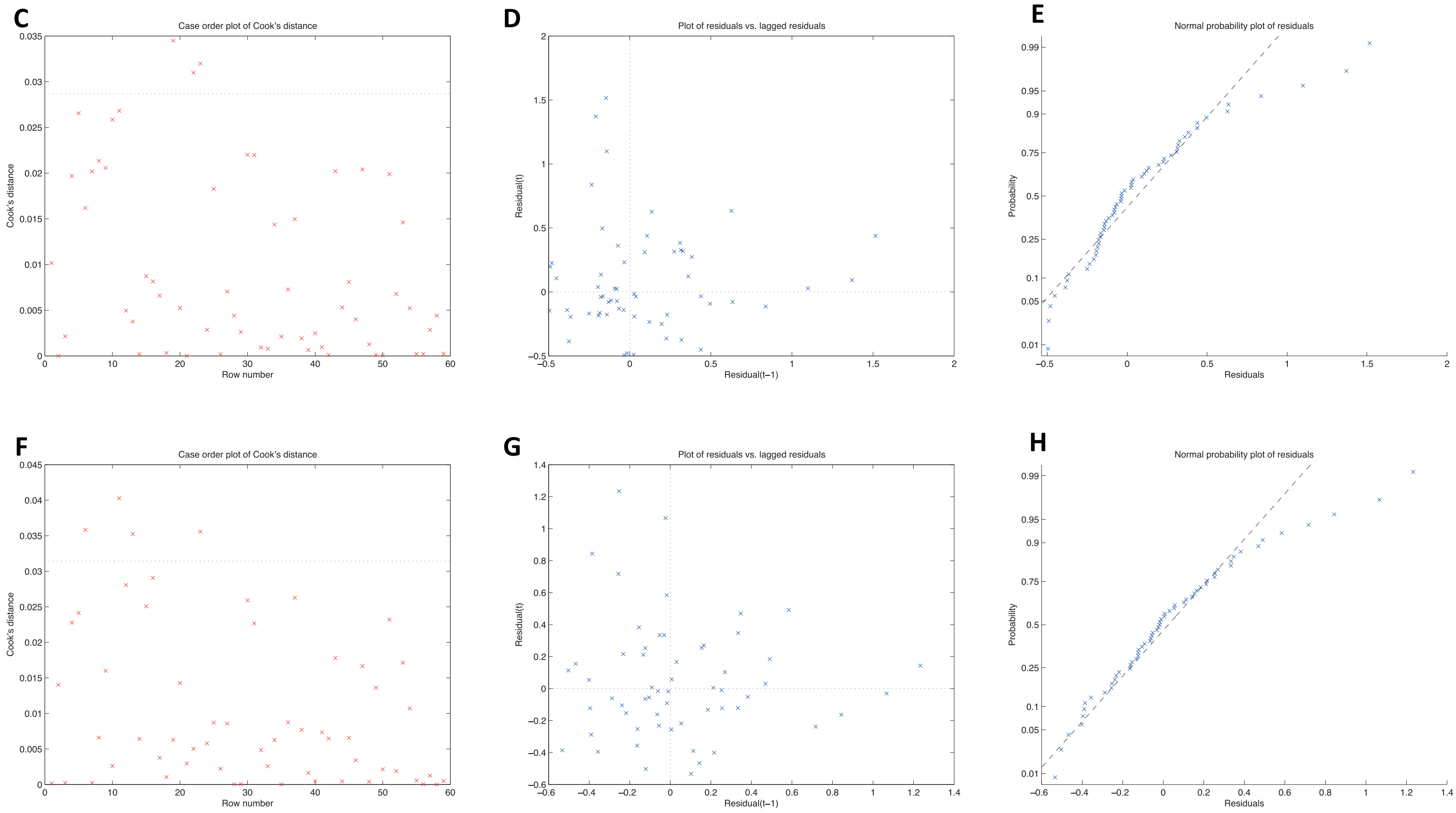

Supplement: S8 Fig — To test whether the normalized displacement and cumulative distance values (dspl/t and cumD/t respectively) presented in Fig 6 (HUVEC) are different between single and collective outgrowth the data were fit with a linear regression model with the response variable being dspl/t or cumD/t and the predictor variable being a categorical variable with three levels: 0 for single outgrowth and 1 for collective outgrowth. The analysis was performed the same way as described in S4 Fig. The results of the fit (coefficient estimates and statistics for the null hypothesis that the coefficients are zero) are shown in (A) for dspl/t and (B) for cumD/t. For dspl/t, p-value = 0.259, while for cumD/t, p-value = 0.013, showing that only cumulative distance is different in collective outgrowth and only at the 0.05 level. The graphs in (C)-(E) and (F)-(H) show some of the model diagnostics for dspl/t and cumD/t respectively, as described in S4 Fig, that validate the linear regression model. (PDF) [file pone.0145210.s008.pdf]
