# Supplementary material for: Walking the Line: A Fibronectin Fiber-Guided Assay to Probe Early Steps of (Lymph)angiogenesis
Source: PLoS One. 2015 Dec 21;10(12):e0145210. doi: 10.1371/journal.pone.0145210 (PMC4686943; doi:10.1371/journal.pone.0145210)
Supplement: S1 Table — A chi-square test was performed to assess whether the percentage of beads that gave rise to single vs. collective outgrowth or to one vs. multiple events per bead were different in the absence or presence of VEGF-A and/or VEGF-C. The observed (obs) numbers of beads under each condition are compared with the expected (exp) numbers, assuming a random distribution across the 4 conditions. For each condition (column) 4 measures are indicated: the chi-square value (chi^2), the residual (res) defined as the difference between observed and expected numbers, the standardized residual (std. res) defined as obs−expexp and the adjusted residual (ads. res) defined as obs−expexp*1−row totaln*1−column totaln, where n is the number of observations, row total the sum of observation values of the respective row and column total the sum of observation values of the respective column. If the chi-square test shows overall statistical significance, large values of standardized or adjusted residuals (typically larger than 2) point to the groups with the statistical difference. The values of the residuals (res) are not as reliable indicators of statistical significance since they depend on the observed numbers. For a detailed description of chi-square statistical analysis refer to Sharpe D, Practical Assessment, Research and Evaluation, 2015, 20(8): 1–10. (PDF) [file pone.0145210.s010.pdf]

suppl. Table 1

			LEC			
			control	VEGF-A	VEGF-C	VEGF-A + VEGF-C
Outgrowth Mode	Single	obs	34	9	36	19
		exp	22.48	24.28	22.93	28.32
		chi^2	5.91	9.61	7.45	3.07
		res	11.52	-15.28	13.07	-9.32
		std. res	2.43	-3.10	2.73	-1.75
		adj. res	3.73	-4.82	4.20	-2.80
	Collective	obs	16	45	15	44
		exp	27.52	29.72	28.07	34.68
		chi^2	4.82	7.85	6.09	2.51
		res	-11.52	15.28	-13.07	9.32
		std. res	-2.20	2.80	-2.47	1.58
		adj. res	-3.73	4.82	-4.20	2.80
Outgrowth Number	One event / bead	obs	29	22	25	21
		exp	22	27	23	25
		chi^2	2.3	0.79	0.1	0.65
		res	7.00	-5.00	2.00	-4.00
		std. res	1.49	-0.96	0.42	-0.80
		adj. res	2.45	-1.63	0.69	-1.34
	Multiple events / bead	obs	13	29	20	27
		exp	20	24	22	23
		chi^2	2.51	0.87	0.11	0.71
		res	-7.00	5.00	-2.00	4.00
		std. res	-1.57	1.02	-0.43	0.83
		adj. res	-2.46	1.66	-0.68	1.34

			HUVEC			
			control	VEGF-A	VEGF-C	VEGF-A + VEGF-C
Outgrowth Mode	Single	obs	56	47	61	32
		exp	55	49	55	36
		chi^2	0.01	0.1	0.62	0.55
		res	0.85	-2.23	5.85	-4.47
		std. res	0.11	-0.32	0.79	-0.74
		adj. res	0.18	-0.50	1.26	-1.11
	Collective	obs	65	61	60	48
		exp	66	59	66	46
		chi^2	0.01	0.08	0.52	0.46
		res	-0.85	2.23	-5.85	2.47
		std. res	-0.10	0.29	-0.72	0.37
		adj. res	-0.18	0.50	-1.26	0.60
Outgrowth Number	One event / bead	obs	13	28	15	15
		exp	19	19	19	14
		chi^2	1.68	3.92	0.86	0.06
		res	-6.00	9.00	-4.00	1.00
		std. res	-1.38	2.06	-0.92	0.27
		adj. res	-1.84	2.77	-1.23	0.34
	Multiple events / bead	obs	65	53	65	44
		exp	59	62	61	45
		chi^2	0.52	1.23	0.27	0.02
		res	6.00	-9.00	4.00	-1.00
		std. res	0.78	-1.14	0.51	-0.15
		adj. res	1.86	-2.74	1.23	-0.34
