# Supplementary material for: Walking the Line: A Fibronectin Fiber-Guided Assay to Probe Early Steps of (Lymph)angiogenesis
Source: PLoS One. 2015 Dec 21;10(12):e0145210. doi: 10.1371/journal.pone.0145210 (PMC4686943; doi:10.1371/journal.pone.0145210)
Supplement: S2 Table — The raw data of normalized displacement and cumulative distance values of single or collective LEC and HUVEC outgrowth in the absence or presence of VEGF-A and/or VEGF-C used in S2 Fig are shown along with the bead and total range and average values (as defined in S2 Fig). (PDF) [file pone.0145210.s011.pdf]

suppl. Table 2

LEC
Beads with multiple events of single outgrowth

		dspl/t					cumD/t				
		values for individual events	bead range	bead range/total range	bead average	bead average/total average	values for individual events	bead range	bead range/total range	bead average	bead average/total average
Bead 1	Event 1<br>Event 2	0.510<br>0.241	0.268	0.351	0.376	1.329	0.893<br>0.667	0.226	0.262	0.780	1.043
Bead 2	Event 1<br>Event 2	0.576<br>0.292	0.284	0.372	0.434	1.534	1.136<br>0.748	0.388	0.450	0.942	1.259
Bead 3	Event 1<br>Event 2	0.177<br>0.159	0.018	0.024	0.168	0.594	0.871<br>0.808	0.063	0.073	0.839	1.122
Bead 4	Event 1<br>Event 2	0.242<br>0.151	0.092	0.120	0.197	0.696	0.958<br>0.743	0.215	0.249	0.851	1.138
Bead 5	Event 1<br>Event 2	0.015<br>0.097	0.083	0.109	0.056	0.198	0.727<br>0.918	0.191	0.222	0.822	1.099
Bead 6	Event 1<br>Event 2	0.222<br>0.038	0.184	0.241	0.130	0.459	0.585<br>0.310	0.275	0.319	0.447	0.598
Bead 7	Event 1<br>Event 2	0.342<br>0.154	0.187	0.245	0.248	0.876	0.512<br>0.476	0.036	0.042	0.494	0.660
Bead 8	Event 1<br>Event 2	0.766<br>0.002	0.764	1.000	0.384	1.357	0.956<br>0.916	0.040	0.046	0.936	1.251
Bead 9	Event 1<br>Event 2	0.500<br>0.087	0.413	0.541	0.293	1.035	0.641<br>0.591	0.050	0.058	0.616	0.824
Bead 10	Event 1<br>Event 2	0.474<br>0.446	0.028	0.037	0.460	1.625	0.807<br>0.701	0.105	0.122	0.754	1.008
Bead 11	Event 1<br>Event 2	0.141<br>0.503	0.361	0.473	0.322	1.138	0.830<br>0.671	0.159	0.184	0.750	1.003
Bead 12	Event 1<br>Event 2	0.401<br>0.404	0.002	0.003	0.403	1.424	0.468<br>0.487	0.020	0.023	0.477	0.638
Bead 13	Event 1<br>Event 2<br>Event 3	0.090<br>0.639<br>0.214	0.549	0.719	0.314	1.110	0.751<br>1.172<br>0.921	0.421	0.488	0.948	1.267
Bead 14	Event 1<br>Event 2<br>Event 3	0.149<br>0.234<br>0.192	0.085	0.111	0.192	0.678	0.902<br>0.760<br>0.771	0.142	0.165	0.811	1.084
Bead 15	Event 1<br>Event 2<br>Event 3	0.399<br>0.182<br>0.303	0.217	0.284	0.295	1.042	0.638<br>0.679<br>0.678	0.041	0.048	0.665	0.889
Total			0.764		0.283			0.862		0.748	

LEC
Beads with multiple events of collective outgrowth

		dspl/t					cumD/t				
		values for individual events	bead range	bead range/total range	bead average	bead average/total average	values for individual events	bead range	bead range/total range	bead average	bead average/total average
Bead 1	Event 1<br>Event 2	0.205<br>0.184	0.021	0.058	0.195	0.783	0.643<br>0.386	0.257	0.576	0.515	0.880
Bead 2	Event 1<br>Event 2	0.092<br>0.106	0.015	0.042	0.099	0.398	0.847<br>0.604	0.242	0.543	0.725	1.239
Bead 3	Event 1<br>Event 2	0.152<br>0.138	0.014	0.039	0.145	0.582	0.858<br>0.520	0.338	0.758	0.689	1.178
Bead 4	Event 1<br>Event 2	0.328<br>0.214	0.114	0.318	0.271	1.088	0.535<br>0.353	0.183	0.410	0.444	0.759
Bead 5	Event 1<br>Event 2	0.165<br>0.158	0.006	0.017	0.161	0.647	0.558<br>0.452	0.106	0.238	0.505	0.863
Bead 6	Event 1<br>Event 2	0.571<br>0.513	0.058	0.162	0.542	2.177	0.639<br>0.693	0.054	0.121	0.666	1.138
Bead 7	Event 1<br>Event 2	0.506<br>0.222	0.284	0.791	0.364	1.462	0.847<br>0.507	0.34	0.762	0.677	1.157
Bead 8	Event 1<br>Event 2	0.211<br>0.212	0.001	0.003	0.212	0.851	0.411<br>0.513	0.101	0.226	0.462	0.790
Total			0.359		0.249			0.446		0.585	

HUVEC
Beads with multiple events of single outgrowth

		dspl/t					cumD/t				
		values for individual events	bead range	bead range/total range	bead average	bead average/total average	values for individual events	bead range	bead range/total range	bead average	bead average/total average
Bead 1	Event 1<br>Event 2	0.061<br>1.039	0.979	0.440	0.550	0.548	0.749<br>1.336	0.587	0.346	1.043	0.789
Bead 2	Event 1<br>Event 2<br>Event 3	0.496<br>1.016<br>1.043	0.546	0.246	0.851	0.849	0.771<br>1.029<br>1.309	0.538	0.317	1.036	0.784
Bead 3	Event 1<br>Event 2<br>Event 3	1.703<br>1.451<br>0.052	1.651	0.743	1.069	1.066	1.725<br>1.459<br>1.019	0.706	0.415	1.401	1.060
Bead 4	Event 1<br>Event 2	0.740<br>1.545	0.805	0.362	1.142	1.139	1.235<br>1.594	0.360	0.212	1.414	1.070
Bead 5	Event 1<br>Event 2<br>Event 3<br>Event 4	0.588<br>2.014<br>1.235<br>0.295	1.718	0.773	1.033	1.030	1.361<br>2.027<br>1.627<br>1.131	0.896	0.527	1.536	1.162
Bead 5	Event 1<br>Event 2<br>Event 3	2.275<br>1.197<br>0.307	1.968	0.886	1.260	1.256	2.300<br>1.210<br>0.600	1.700	1.000	1.370	1.036
Total			2.223		1.003			1.700		1.322	

HUVEC
Beads with multiple events of collective outgrowth

		dspl/t					cumD/t				
		values for individual events	bead range	bead range/total range	bead average	bead average/total average	values for individual events	bead range	bead range/total range	bead average	bead average/total average
Bead 1	Event 1<br>Event 2	0.553<br>1.225	0.672	0.463	0.889	1.040	0.709<br>1.488	0.779	0.589	1.098	1.063
Bead 2	Event 1<br>Event 2	1.741<br>0.830	0.911	0.628	1.285	1.503	1.767<br>1.092	0.675	0.511	1.429	1.384
Bead 3	Event 1<br>Event 2<br>Event 3	1.571<br>1.264<br>1.041	0.531	0.365	1.292	1.511	1.588<br>1.281<br>1.138	0.449	0.340	1.336	1.293
Bead 4	Event 1<br>Event 2	0.819<br>0.339	0.480	0.331	0.579	0.677	1.240<br>0.819	0.421	0.319	1.030	0.997
Bead 5	Event 1<br>Event 2	0.827<br>0.457	0.370	0.255	0.642	0.751	0.835<br>0.614	0.221	0.167	0.724	0.701
Bead 6	Event 1<br>Event 2	0.289<br>0.638	0.349	0.240	0.463	0.542	0.446<br>0.669	0.223	0.168	0.557	0.540
Bead 7	Event 1<br>Event 2	0.469<br>0.766	0.297	0.205	0.618	0.723	0.728<br>1.076	0.348	0.263	0.902	0.873
Total			1.452		0.855			1.321		1.033	
